# A nanobody-stem cell platform targeting innate and adaptive immune axis in the tumour microenvironment

**DOI:** 10.1016/j.ebiom.2026.106122

**Published:** 2026-01-17

**Authors:** Ioulia Vogiatzi, Amelia Lehmann, Chuang Liu, Lucia Moreno-Lama, Yoshinori Kajiwara, Nobuhiko Kanaya, Olivier Zwaenepoel, Ali Salehi Farid, Uk-Jae Lee, Filippo Rossignoli, Mohammad Rashidian, Hiroaki Wakimoto, Jan Gettemans, Khalid Shah

**Affiliations:** a Center for Stem Cell and Translational Immunotherapy, Brigham and Women's Hospital, Harvard Medical School, Boston, MA, 02115, USA; b Department of Neurosurgery, Brigham and Women's Hospital, Harvard Medical School, Boston, MA, 02115, USA; c Department of Biomolecular Medicine, Faculty of Medicine and Health Sciences, Ghent University, Belgium; d Harvard Stem Cell Institute, Harvard University, Cambridge, MA, 02138, USA; e Dana-Farber Cancer Institute, Harvard Medical School, Boston, MA, 02115, USA; f Department of Neurosurgery, Massachusetts General Hospital, Harvard Medical School, Boston, MA, 02114, USA

**Keywords:** Nanobodies, Stem cells, Macrophages, T cells, Immunotherapy

## Abstract

**Background:**

Tumour associated macrophages (TAMs) and exhausted T cells are dominant in the immuno-suppressive tumour microenvironment (TME) and pose a challenge to effective cancer immunotherapy in solid tumours.

**Methods:**

In this study, we immunised llamas and generated monovalent and biparatopic nanobodies (Nbs) against colony stimulating factor 1 receptor (CSF-1R) and programmed death receptor 1 (PD-1) to re-educate TAMs and overcome T cell exhaustion, respectively, in the TME. To circumvent short systemic half-life and low peak concentrations of Nbs in the TME, we developed a platform of allogenic off-the-shelf stem cells (SC) releasing biparatopic PD-1 and CSF-1R Nbs and tested its efficacy in different mouse models.

**Findings:**

Nbs targeting CSF-1R and PD-1 inhibited the CSF1/CSF-1R and PD-1/PD-L1 pathways, respectively, with biparatopic Nbs demonstrating superior efficacy and functionality compared with their monovalent counterparts. Locoregional SC mediated release of biparatopic CSF-1R and PD-1 Nbs, reduced tumour growth by increasing T cell numbers, enhancing T cell activation, and by shifting the macrophage polarisation towards a pro-inflammatory phenotype. Moreover, the presence of both Nbs improved dendritic cells (DCs) activation within the TME. Finally, we show that biocompatible gel encapsulated SC releasing Nbs against PD-1 and CSF-1R have therapeutic efficacy in a highly immunosuppressive glioblastoma model post-tumour resection.

**Interpretation:**

Taken together, our findings establish a cell-based Nb platform targeting both innate and adaptive immune axis within the TME, which has the potential to facilitate treatment of solid cancers that are otherwise refractory to conventional immunotherapies.

**Funding:**

This study was mainly supported by Institutional Funds (K.S). A part of *in vivo* Nb characterisation was supported by 10.13039/100000002NIH grant R01-CA285519 (K.S.) and the Nb dye conjugation and modelling was supported by 10.13039/100000002NIH grant R01-AI165666 (M.R).

**Research in context**

**Evidence before this study**

To identify relevant studies prior to this investigation, we systematically searched PubMed with keywords as “tumour associated macrophages”, “CSF-1R”, “macrophage mediated T-cell exhaustion”, “nanobody treatment and half-life” and “cancer microenvironment”. For antibody administration systemically, the dosages were in accordance with previously reported studies and searched as “anti-PD1 and anti-CSF1R targeting”. Additionally, we investigated the ClinicalTrials.gov to review clinical trials that combine PD-1 with CSF-1R targeting. Inclusion criteria encompassed preclinical studies that examined the roles of TAMs in T cell exhaustion and tumour support, and the intertwined roles of TAMs into immune cell infiltration in solid tumours, as well as interventions targeting these pathways, and trials with published results. Studies that were not directly addressing immune modulation in the TME were excluded. Altogether, our review of *in vitro*, *in vivo* pre-clinical studies and clinical studies, suggested that blockade of CSF-1R or PD-1 pathways individually can modestly improve anti-tumour immunity, however with limited success in certain resistant solid tumour types, and increased toxicity, indicating limited translational possibility. Meta-analyses indicated that combined immune checkpoint blockade and TAM modulation hold potential but require more effective delivery methods to enhance therapeutic efficacy.

**Added value of this study**

Our study introduces a cell-based nanobody platform designed to simultaneously target innate and adaptive immune suppression within the TME. While previous approaches relied mainly on systemic administration of monoclonal antibodies, here we demonstrate the development of biparatopic nanobodies against CSF-1R and PD-1, which exhibit excellent functional activity. We utilised an allogenic stem cell platform to release these nanobodies locally, overcoming limitations related to short systemic half-life, and boost tumour penetration. We showed increased tumour suppression, T cell activation, macrophage re-polarisation and achieved the establishment of a more immune active niche, in challenging models such as glioblastoma, a tumour type resistant to existing immunotherapies. These results provide a conceptual and translational advancement with potential to improve outcomes in resistant solid tumours.

**Implications of all the available evidence**

Our findings along with the existing research studies underscore the therapeutic potential of combination treatment targeting TAMs and exhausted T cells within the TME. Our proposed platform offers an avenue to enhance tumour immune reactivation, particularly in “cold” solid tumours. This could lead to more effective and localised immunotherapies with reduced systemic toxicity. Future research could focus on exploring combination strategies with existing therapies such as chemotherapy, radiotherapy, and other immune modulatory agents. Overall, our study has implications in the field of precision immunotherapy by harnessing bioengineered cell platforms to modulate multiple immune pathways simultaneously, thereby expanding the therapeutic landscape for challenging cancers.

## Introduction

Despite continuous efforts to develop immune based therapies, currently approved Immunotherapeutics have not yet yielded the anticipated benefits in solid primary and metastatic tumours.^1^ Although initial responses are often observed, the complex tumour microenvironment (TME) eventually evades therapies, resulting in sustained suppressive immune environment and treatment resistance.^2^ Therefore, there is an unmet need to develop the next generation of biological tools and therapeutic strategies that more effectively target the TME.

Monoclonal antibodies (Abs) have shown clinical success and are approved for several cancer types, however restraints such as tumour penetration and therapy resistance remain challenging in the majority of solid tumours.^3^, ^4^, ^5^ Nanobodies (Nbs), also known as variable domain of heavy chain Abs or single-domain Abs (sdAbs), are small antibody fragments derived from heavy chain-only Abs found in camelids.^6^ They offer a stable therapeutic modality with high binding efficacy against the desired epitope.^7^ In addition, their small size, ease of genetic manipulation via cDNA, capacity for expression and secretion by several cell types, in contrast to conventional Abs, facilitate their use and administration.^6^ Biparatopic Nbs which link two Nbs recognising distinct epitopes on the same target, have higher binding efficacy and superior therapeutic functionality as compared to their monovalent counterparts.^8^ Dual binding of biparatopic Nbs increases resistance to proteolytic degradation and enhances the overall Nb stability. Moreover, biparatopic Nbs enable crosslinking of the target molecules and induce conformational changes^9^ which lead to modulating target activity, through mechanisms such as receptor clustering, thereby conferring improved therapeutic efficacy.^9^, ^10^, ^11^

Recent immunotherapeutic approaches have increasingly focused on targeting the tumour associated macrophages (TAMs) in solid tumours, as they constitute the most abundant non-malignant cell types within the TME.^12^^,^^13^ Cancer cell-derived CSF1 interacts with CSF-1R on TAMs, driving their polarisation towards a pro-tumoral phenotype that promotes cancer progression.^14^ Although CSF-1R blockade initially showed promise in suppressing tumour growth across multiple cancer types, its use as monotherapy often leads to therapy resistance and cancer reoccurrence.^15^, ^16^, ^17^ Notably, combining CSF-1R blockade with radiotherapy has been shown to overcome CSF-1R resistance.^18^ Furthermore, pro-tumoral TAMs and macrophages (MΦ) overexpress PD-1 and PD-L1 on their surface,^19^^,^^20^ and blockade of this pathway has the potential to repolarize TAMs towards an anti-tumoural phenotype and restore their phagocytotic function.^21^^,^^22^

Immune checkpoint inhibitors (ICIs) targeting the PD-1/PD-L1 pathway have presented significant clinical success in certain cancers, such as melanoma.^23^ However, their efficacy in most solid tumours remains limited due to the highly complex and immune suppressive TME.^24^^,^^25^ Beyond TME related barriers, systemic administration of ICIs is associated with immune-related toxicities.^26^^,^^27^ Many of the immunomodulators agents also exhibit short systemic half-life and low peak concentrations restricting their accumulation to therapeutically effective levels within the TME.^28^ Moreover, currently used therapeutic Abs, such as Ipilimumab, a CTLA4 monoclonal Ab approved for primary and metastatic melanoma,^29^ are associated with off-target toxicities and limited tumour penetration.^30^^,^^31^

To circumvent these issues, we and others have explored the local delivery of therapeutics using stem cells (SCs). In particular, we have shown that allogeneic “off-the-shelf” mesenchymal SC exhibit potent pathotropic migratory properties,^32^, ^33^, ^34^ rendering them attractive for use as targeted delivery vehicles in cancer therapy. Furthermore, SCs are attractive for therapeutic engineering as they exhibit high metabolic activity and thus strong expression of transgenes *in vitro* and *in vivo* and notably low immunogenicity,^35^, ^36^, ^37^ which allows them to evade detection and destruction by the host's immune system.^38^ More specifically, SCs exhibit very low levels of Major Histocompatibility Complex (MHC) I and II, reducing recognition by cytotoxic T cells. In addition, SCs secrete immunomodulatory molecules that further dampen immune responses and promote immune tolerance.^38^ This immune-privileged profile permits repeated administration and long-term applications without eliciting significant immune responses,^39^ a characteristic particularly advantageous in cancer therapies, where minimising immune interference is crucial for effective treatment outcomes and long-term efficacy. Collectively, these attributes along with their “off-the-shelf” availability render SC as targeting therapeutic delivery vehicles and strengthen the rationale for their use as targeted delivery vehicles for the local release of Nbs.

In this study, our goal was to develop tools combined with tumour homing delivery vehicles, that concurrently target the CSF-1/CSF-1R and PD-1/PD-L1 pathways to address heterogeneous TME in solid tumours. To this end, we immunised llamas with the purified extracellular domain of CSF-1R and PD-1 proteins, selected Nbs specific to these target receptors. We characterised the CSF-1R and PD-1 Nbs, engineered SCs to release biparatopic Nbs and assessed their mechanism-based efficacy *in vitro* and in multiple murine tumour models.

## Methods

### Nbs generation, production and purification

Llamas were immunised with recombinant extracellular domains of mouse PD-1 and mouse CSF-1R. mRNA was extracted from B cells, reverse transcribed, and VHH cDNA sequences were cloned into a phage display vector and the library was constructed. Following panning against target proteins, specific binders were enriched and selected. Selected Nbs were cloned into the pMECS phagemid vector with HA and His tags and expressed in E. coli WK6 cells. Cultures were grown in ampicillin (CORNING, 61238 R H) containing media and Nb expression induced with IPTG (Sigma, I6758-1G) at OD600 ≈ 0.6, followed by overnight incubation at 28 °C. Bacteria were centrifuged, and the pellets were resuspended in TES buffer (0.2 M Tris base, 0.5 mM EDTA (Boston Bioproducts, BM-150) and 0.5 M sucrose (Fisher Bioreagents, BP220-1) in dH_2_O, pH 8) and placed on 4 °C for at least 1 h while rotating. Finally, TES/4 buffer was added, and the mix was incubated ON at 4 °C, while rotating, to break the bacterial periplasm. The mix was centrifuged at maximum speed for an hour and the supernatant containing the periplasmic extracts and the Nbs was collected.

Each Nb was purified manually, with gravity purification column (Bio-Rad, 7316214), using Ni-NTA agarose beads (QIAGEN, 30210). The resin was washed with 15 mM imidazole (Sigma–Aldrich, I2399-100G) in 1× PBS (Gibco, 14040133) to prevent unspecific binding, and the Nbs were eluted with 300 mM imidazole in 1× PBS. Each Nb elution was concentrated and reconstituted with 1× PBS to remove the imidazole. The purity was verified by SDS electrophoresis performed in precast 4–20% acrylamide gel (Bio-Rad, 4561093). The gel was stained with Coomassie Brilliant Blue R-250 Staining Solution (Bio-Rad, 1610436) and de-stained with Coomassie Brilliant Blue R-250 Destaining Solution (Bio-Rad, 1610438).

### Virus generation and SC engineering

Lentiviral packaging (LV- GFP, and LV monovalent or biparatopic Nbs) was carried out by transfection of 293 T cells, and target cells were transduced with lentiviral vectors in Opti-MEM medium (Gibco, 2177672) supplemented with protamine sulphate (2 μg/ml). For bioluminescence imaging (BLI), cells were transduced with LV-GFP-Firefly luciferase (LV-GFP-Fluc). Cells were sorted using a BD FACS Aria Fusion cell sorter.

### Cell culture

293 T cell line (ATCC), melanoma cancer cell line UV2 (provided by J. Sarkaria, Mayo Clinic, Rochester) and murine macrophage cell line RAW264.7 (ATCC) were cultured in DMEM medium with high glucose (Gibco, 11504496), 10% Foetal Bovine Serum (FBS) (Gibco, 10082147) and 1% penicillin/streptomycin (P/S) (Gibco). The murine adipose mesenchymal stem cells (SCs) were cultured in DMEM with low glucose (Gibco, 11885084), 15% FBS, 1% l-Glutamine (Gibco, 25030081), 1% non-Essential Amino Acids (NEAA) (Gibco, 11140050) and 1% P/S. SCs were purchased by Cyagen, and are derived from C57BL/6 mouse adipose tissue. These SC have strong proliferation capabilities and can survive and withstand multiple rounds of cell culture passaging without affecting their quality. TC1 cells (kindly provided by Dr. Pittet, MGH, Boston) were cultured in RPMI 1640 + GlutaMAX (Gibco, 61870036), 10% FBS and 1% P/S. The cells were incubated at 37 °C, in presence of 5% CO_2_. Cell lines were regularly tested for mycoplasma prior to experiments using a mycoplasma PCR kit (30-1012 K, American Type Culture Collection [ATCC]).

### *In vivo* mouse experiments

All *in vivo* experimental protocols were approved by the Subcommittee on Research Animal Care at Brigham and Women's Hospital (2017N000015, 2017N000017, 2019N000204, 2021N000282). All animals were maintained under identical conditions, to minimise confounding effects, and their health status was regularly evaluated. Animals were randomly allocated to cages and experimental groups with the aim of achieving comparable average tumour signal per group. Readouts were unbiased as the tumour sizes were based on the luminescence signal. All statistical analysis were done with n ≥4 and all mice received pre- and post-surgical care, including analgesics, in accordance with the Subcommittee of Research Animal Care in Brigham and Women's Hospital. Animals were housed five per cage, and animals that died or were euthanised due to technical or surgical complications, and for ethical reasons were excluded. These exclusion criteria were defined prior to each experiment. All *in vivo* experiments used humane endpoints, following euthanasia using CO_2_. *In vivo* experiments were performed on immunocompetent mice to determine the effect of treatment on the immune system. In all *in vivo* experiments SC-GFP cells were used as a control. Mouse metastatic lung cancer model TC1-GFP-Fluc was established as previously described,^40^, ^41^ n = 5. The tumour growth was monitored by *In Vivo* Imaging System (IVIS). In the control group (SC-GFP) one mouse was not included in the imaging on day 4 due to worsening body conditions, which then reached endpoint on day 7. A subcutaneous melanoma model was established using UV2 cells (1 × 10^6^ cells/mouse). On day five and eight post tumour implantation, therapeutic SC-BNbPD1 and/or SC-BNbCSF1R (1∗10^6^ cells/mouse) were implanted intratumorally in female C57BL/6 (6–8 weeks of age). Tumour growth was monitored by calliper measurements, n = 7, (SC-GFP = 6) and by IVIS. A GBM CT2A resection model was established using immunosuppressive CT2A-FmC cells (1.5 × 10^5^ cells/mouse). On day seven, partial tumour resection was performed, followed by placement of encapsulated therapeutic SC-BNbPD1 and/or SC-BNbCSF1R (total 1 × 10^6^ cells/mouse) in the resection cavity in female C57BL/6 mice (6–8 weeks of age) as described previously.^42^ Tumour growth was monitored by IVIS, n = 4, (SC-BNbPD1 = 5). The reduced group sizes (n = 4) were due to data exclusion following technical and surgical complications. Surviving mice from these GBM *in vivo* studies were euthanised, and major organs were collected and sectioned. Haematoxylin and Eosin staining was performed as described below, and the pathological assessment was conducted.

### *In vivo* ELISA studies

A subcutaneous melanoma model was established by implanting UV2 cells (1 × 10^6^ cells/mouse) in immunocompetent C57BL/6 mice (6–8 weeks of age). On day five AbPD1 (200 µg/mouse) and AbCSF1R (600 µg/mouse) were administered intravenously via the tail vain. Control mice received PBS injections. Additionally, UV2 cells (1 × 10^6^/mouse) and therapeutic SC-BNbPD1 or SC-BNbCSF1R (1 × 10^6^/mouse) were co-implanted in nude mice (6–8 weeks). Immunodeficient mouse models were used because, in immunocompetent mice, tumours were nearly completely eliminated within seven days of the SC-BNbPD1 and SC-BNbCSF1R implantation, preventing the collection of sufficient tissue for further analysis. Seven days post administration of Abs and implantation of SC, tumours were collected, homogenised and lysed. Samples were analysed for IgG2a (ThermoScientific, Cat# 88-50510-22) to detect the antibodies, and for VHH (GenScript, Cat# L01033) to detect the Nbs, using ELISA kits. Plates were read at 450 nm.

### Statistical analysis

Statistical analysis was performed using GraphPad Prism software. Student's t test and one- or two-way ANOVA analysis were used to assess the significance of differences between groups. Data variability was measured with mean and SD. The Mantel–Cox (log-rank) test was used for Kaplan–Meier survival analyses. This study is a proof-of-concept study. Experiments were performed with a minimum of three replicates to ensure sufficient statistical power for comparisons. Differences were considered significant at ∗P < 0.05, ∗∗P < 0.01, ∗∗∗P < 0.001, and ∗∗∗∗P < 0.0001.

### Role of funders

Funders had no role in study design, data collection, data analyses, interpretation, or writing of the report.

## Results

### Higher CSF-1R expression is associated with reduced patient survival and increased PD-1 expression

**Fig. 1:**
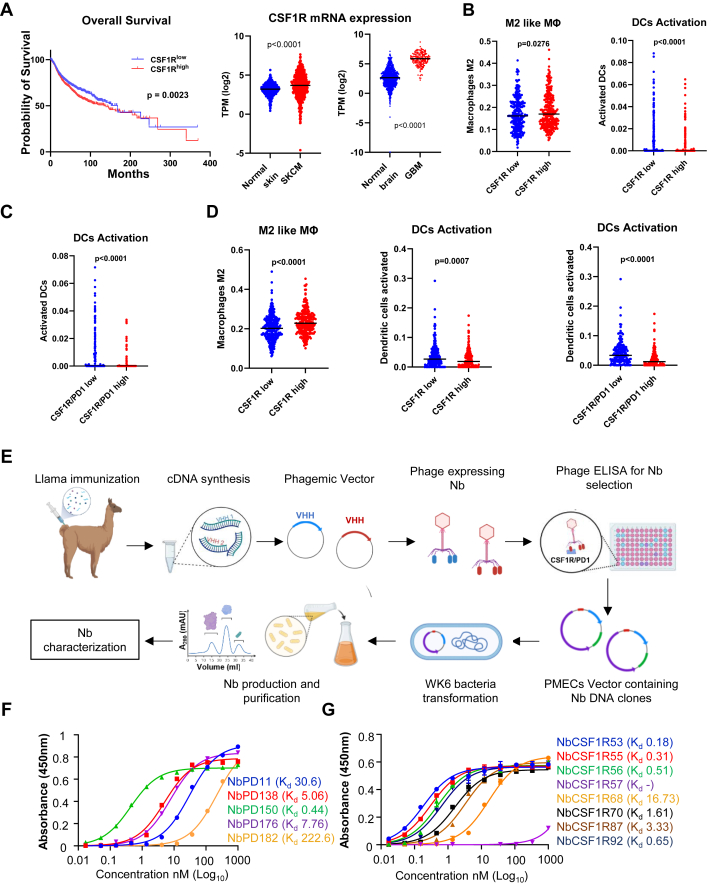
**Development of monovalentNbsagainst PD-1 and CSF-1R.** A) TCGA analysis presenting the overall survival (left) in pan-cancer types at the highest and lowest expression levels of CSF-1R (Mantel–Cox test survival analysis). CSF-1R mRNA expression levels (right) in healthy skin and brain compared to melanoma and GBM, respectively. B, C, D) M2 MΦ levels and DCs activation in SKCM and lung cancer in the different levels of CSF-1R and PD-1 expression (Student t-test). E) Schematic representation of llama immunisation and phage ELISA for the Nb preselection. F, G) Ability of different PD-1 and CSF1-R Nb clones to bind PD-1 and CSF1-R recombinant protein, respectively. The binding was performed in duplicates and assessed based on the absorbance at 450 nm, using a plate reader, and the affinity (Kd) was assessed using GraphPad Prism one-site Specific Binding analysis. (∗P < 0.05, ∗∗P < 0.01, ∗∗∗P < 0.001, and ∗∗∗∗P < 0.0001).

To assess how CSF-1R overexpression affects the TME and its relevance to patient outcomes, various tumours from The Cancer Genome Atlas (TCGA) were analysed and stratified into high and low CSF-1R expression groups. Upregulated CSF-1R expression was associated with decreased patient survival in pan-cancer analysis as well as in selected cancer-specific subtypes (Mantel–Cox test) (Fig. 1A, Figure S1A). CSF1R mRNA expression was higher in both skin cutaneous melanoma (SKCM) and glioblastoma (GBM), compared with healthy tissue (Fig. 1A). In SKCM, the CSF-1R high group showed increased proportion of M2-like MΦ (t-test, P = 0.0276) and decreased number of activated DCs (t-test, P < 0.0001) (Fig. 1B). Simultaneous overexpression of CSF-1R and PD-1 was also associated with significantly reduced numbers of activated DCs (t-test, P = 0.0001) (Fig. 1C). TCGA analysis of lung adenocarcinoma revealed similar results (Fig. 1D). In both GBM and melanoma, the expression of PDCD1 expression was higher relative to normal tissue, and the expression of PDCD1 and CSF1R were positively correlated (Figure S1B, C). In GBM, the proportion of M2 MΦ was particularly high, suggesting that therapeutic inhibition of CSF1R could be effective in this context (Figure S1D). Additionally, M2-like MΦ and monocytes were significantly enriched in CSF1R-high GBM compared with CSF1R low GBM. In parallel, CSF1R-high GBM tumours had reduced activation of immune populations, such as T follicular helper cells, indicating an overall non immunogenic niche (Figure S1D). Together these findings suggest that concurrent overexpression of PD-1 and CSF-1R pathways may act in concert to drive the suppressive TME, ultimately contributing to poor patient outcomes. Consequently, we sought to assess the therapeutic impact of blocking these pathways using Nbs, with the aim of reprogramming, immune milieu towards an anti-tumour phenotype.

### Selection and characterisation of anti-PD-1 and anti–CSF–1R Nbs

To generate PD-1 and CSF-1R specific Nbs clones, llamas were immunised with the extracellular ectodomains (ECD) of mouse PD-1 and CSF-1R, respectively. Llama lymphocytes were isolated and used to construct phage libraries for both targets, PD-1 and CSF-1R. Mouse specific Nbs were selected through three panning rounds after which 100 potent clones were selected and subjected to phage ELISA against purified mouse PD-1 and CSF-1R. The strongest binders were selected for further characterisation (Fig. 1E). To screen CSF-1R Nb clones, periplasmic ELISA was performed to assess the level of protein production and total periplasmic binding (Figure S2A). Following this, the complementarity-determining regions (CDR)3 were analysed, leading to the selection of 8 unique Nb clones. These clones were then characterised to identify clones with the highest binding affinity (K_d_). Individual Nbs against PD-1 or CSF-1R were purified and assessed in binding assays using immobilised PD-1 or CSF-1R ECDs. Among the purified clones, Nb-PD138, Nb-PD150 as well as Nb-CSF1R53, Nb-CSF1R55, NbCSF1R56 NbCSF1R92 and Nb-CSF1R70, demonstrated the most favourable binding affinities to immobilised PD-1 and CSF-1R ECDs, respectively, with K_d_ < 10 nM (±0.05 nM) (Fig. 1F and G). CSF-1R specific clones were further tested for binding on bone marrow derived macrophages (BMDM), and their ability to compete with a commercially available neutralising antibody against CSF-1R for epitope occupancy, was tested (Figure S2B). Among these, NbCSF1R70 showed the most effective competition with the CSF-1R antibody, compared with other Nb clones (Figure S2B). These results demonstrate the successful generation of high binding affinity Nbs targeting CSF-1R and PD-1 Nbs.

### Purified anti-PD1 and anti-CSF1R Nbs induce T cell activation and block macrophage proliferation *in vitro*, respectively

**Fig. 2:**
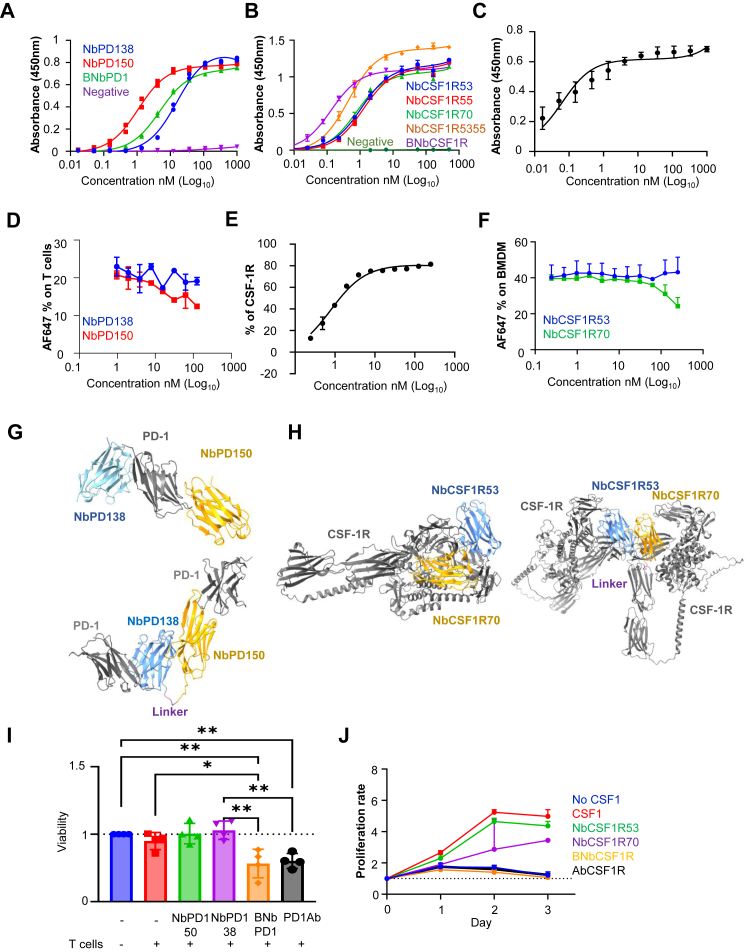
**Generation and functional characterisation of the biparatopic Nbs against PD-1 and CSF-1R.** A, B) Binding affinity of the monovalent and biparatopic Nbs against PD-1 and CSF1-R ECD. The binding assay was performed in duplicates and assessed based on the absorbance at 450 nm, using a plate reader, and the affinity (Kd) was assessed using GraphPad Prism one-site Specific Binding analysis. C, E) Binding capacity of the conjugated Nb (n = 2 for BNb-PD1, binding capacity assessed based on absorbance at 450 nm using a plate reader, n = 3 for BNb-CSF1R, binding capacity assessed using flow cytometry). D, F) Competition Assays between the two monovalent Nbs against their respective targets (n = 2 for monovalent Nbs against PD1 and n = 3 for monovalent Nbs against CSF1R). G, H) Protein docking of Nbs against PD-1 and CSF-1R. I) *in vitro* tumour cell viability assay using T cells isolated from tumour bearing mice. CT2A tumour cells were incubated 1:10 with T cells (control) or T cells and 500 nM of Nb-PD138, Nb-PD150 and BNb-PD1 (experimental), n = 4. The viability was measured by the Luciferase activity. Significance was measured by one-way ANOVA analysis and Tukey's multiple comparisons, where P < 0.05 is significant. As a positive control a PD-1 neutralising Ab was used at the same concentration as the Nbs. J) Proliferation of BMDM in presence of Nb-CSF1R53, Nb-CSF1R70 and BNb-CSF1R Nbs, n = 3. As positive control AFS98 was used. The significance was calculated using one-way ANOVA analysis, P < 0.05. (∗P < 0.05, ∗∗P < 0.01).

PD-1 and CSF-1R proteins are known to dimerise at the cell surface prior to their internalisation and downstream signalling via their respective pathways.^43^^,^^44^ Therefore, biparatopic Nbs have the advantage of more effectively blocking dimerised receptor conformations than monovalent Nbs.^8^^,^^11^ On this basis, we created highly specific biparatopic Nbs by fusing the cDNAs of two monovalent Nbs via a nucleotide sequence encoding an SGGGS linker. The strongest monovalent binders; NbPD150, NbPD138, NbCSF1R53, NbCSF1R55 and NbCSF1R70, were selected to generate the biparatopic constructs NbPD1-5038 (BNbPD1), NbCSF1R5355 and NbCSF1R5370. For CSF1R targeting, NbCSF1R5370 was chosen since NbCSF1R70 exhibited the highest receptor occupancy, among all monovalent clones, followed by NbCSF1R53 (Figure S2B). The binding capacity of each biparatopic Nb against PD-1 and CSF-1R was evaluated using immobilised ECD (Fig. 2A and B). Biparatopic CSF-1R Nbs displayed significantly enhanced binding compared with their monovalent counterparts. As NbCSF1R5370 (BNb-CSF1R) showed superior binding efficacy than Nb-CSF1R5355, all subsequent experiments were performed with BNb-CSF1R. In contrast, Nb-PD150 exhibited stronger binding than BNb-PD1 in ECD assays, prompting further functional analyses to determine whether BNb-PD1 conferred superior biological activity (Fig. 2A, I). Given that biparatopic Nbs consist of two monovalent units binding distinct epitopes on the same receptor, competition assays were performed. Nb-PD150 and Nb-CSF1R70 were conjugated with Alexa Fluor 647 which was confirmed by SDS page electrophoresis (Figure S3A, C). Fluorescence microscopy indicated effective binding of Nb-PD150-AF647 to T cells, and of Nb-CSF1R70-AF647 to both BMDMs and immortalised MΦ cell line RAW264.7-GFP (Figure S3B, D). The binding affinities were comparable to their unconjugated counterparts (Fig. 2C and E). Competition assays showed that increasing concentrations of Nb-PD138 did not interrupt the binding of Nb-PD150 to PD-1 on T cells, and that Nb-CSF1R53 did not interfere with the Nb-CSF1R70 binding to CSF-1R on the surface of BMDMs (Fig. 2D and F). Protein modelling studies further supported biparatopic binding of distinct epitopes on the respective targets. More specifically, Nb-PD138 was predicted to bind on 9-WLTVS-13 epitope, while Nb-PD150 was predicted to bind 100-AK-101 region. For CSF-1R, Nb-CSF1R53 binding was predicted to interact with the Y236 epitope via its H3 paratope (H3) and PWRGFIIRK via its H2 paratope (H2) while Nb-CSF1R70 was predicted to dock to 308PSIQ (H3) and 601ADEKEALMSELKIMSHL helix (H2) (Fig. 2G and H).

To assess functional activity, tumour cells and T cells were co-cultured and incubated with monovalent and biparatopic PD-1 Nbs. A significant reduction of tumour cell viability was observed in the presence of BNb-PD1, comparable to that achieved with a commercial PD-1 neutralising Ab, whereas monovalent Nb-PD150 or Nb-PD138, showed no effect at 500 nM (One-Way ANOVA, Tukey's multiple comparison test) (Fig. 2I). The effect of BNbPD1 was dose-dependent as treatment at 100 nM was ineffective (S4A). Moreover, no change in T cell viability was detected when incubated with different doses of Nb-PD150, Nb-PD138 or BNbPD1, indicating the lack of T cell toxicity (Figure S4B). Together, these results indicate that BNb-PD1 effectively induces T cell activation by blocking PD-1 resulting in enhanced T cell-mediated tumour cytotoxicity. Similarly, the functional impact of CSF-1R blockade was assessed by comparing BNb-CSF1R with Nb-CSF1R53 and Nb-CSF1R70 in MΦ polarisation assay. BMDMs treated with BNb-CSF1R showed reduced proliferation comparable to that observed with the commercial anti–CSF–1R Ab. This effect was greater than that achieved with monovalent Nbs, indicating that BNb-CSF1R effectively inhibits CSF-1-induced MΦ proliferation (Fig. 2J). These findings show that biparatopic CSF-1R and PD-1 Nbs exert potent and target-specific functional effects on MΦ and T cells, respectively, *in vitro*, consistent with their intended on-target function.

### Engineered SC secrete BNb-PD1 and BNb-CSF1R and alter T cell and MΦ phenotypes *in vitro*

**Fig. 3:**
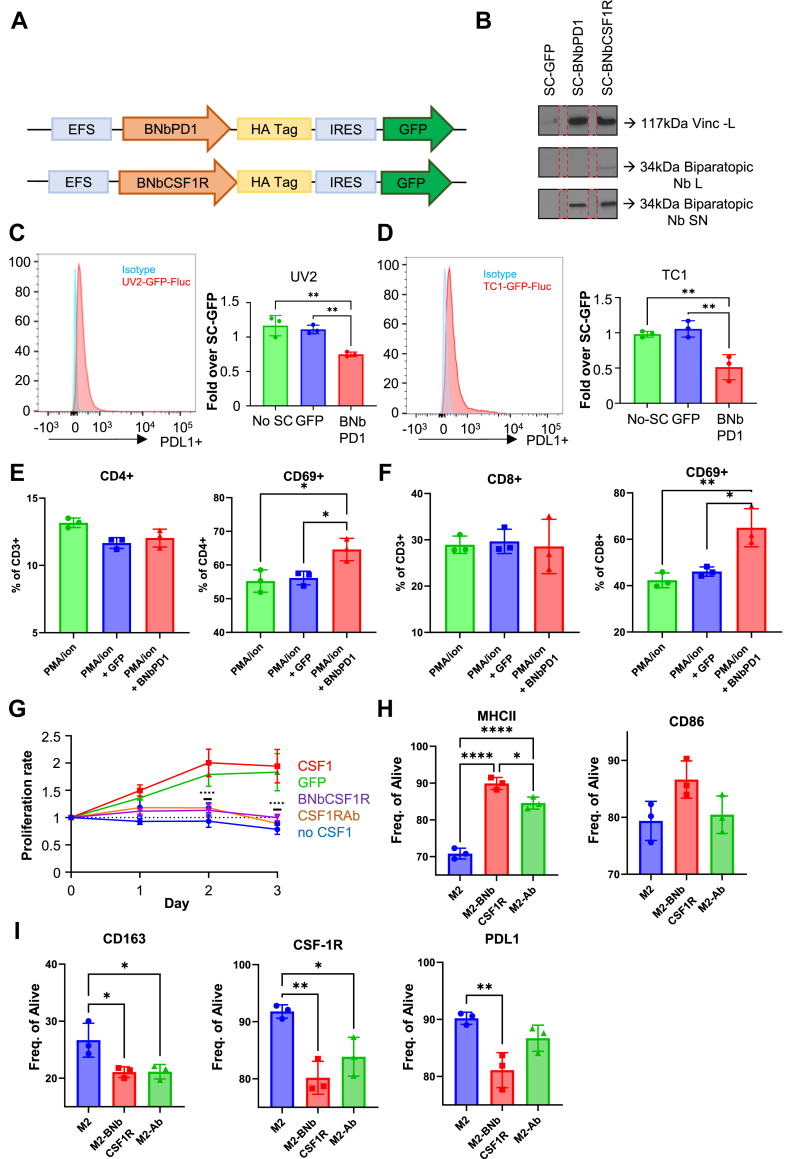
**Engineering SC to secrete BNb-PD1 and BNb-CSF1R and evaluation of their functionality in vitro.** A) Schematic representation of the vectors containing the monovalent and biparatopic Nbs. B) Western blot of SC secreting Nb lysates (L) and supernatant (SN) to verify the Nb secretion. The Nbs were detected with anti HA Tag. Vinculin was used as loading control for the lysates. C, D) *in vitro* TC1-GFP-Fluc and UV2-GFP-Fluc viability assay using T cells isolated from tumour bearing mice implanted with TC1-GFP-Fluc and UV2-GFP-Fluc respectively. The tumour cells were incubated 1:20 with T cells and SC-GFP encapsulated in a gel (control) or T cells and SC-BNb-PD1 (experimental), n = 3. The viability was measured after 48 h based on the Luciferase activity. Significance was measured by one-way ANOVA analysis, where P < 0.05. E, F) graphs showing T cell activation in the total splenocyte population, when splenocytes are incubated with SN from SC-BNbPD1, n = 3 (significance measured by one-way ANOVA analysis with Tukey's multiple comparisons test). G) BMDM growth in presence of SC-BNbCSF1R supernatant, n = 3, (significance measured with Two-Way ANOVA, Tukey's multiple comparisons). H, I) Graphs showing the expression of ΜΦ polarisation markers, after M2 like BMDM have been incubated for 24 h with SN from SC-BNbCSF1R or anti-CSF-1R commercial Ab, clone AFS98, n = 3 (significance measured by one-way ANOVA with Tukey's multiple comparisons) (∗P < 0.05, ∗∗P < 0.01, ∗∗∗P < 0.001, and ∗∗∗∗P < 0.0001).

To enable effective delivery of Nbs in the TME, mesenchymal stem cells (SCs) were engineered to locally secrete biparatopic Nbs. The cDNA encoding each biparatopic Nb fused to a Flt3l secretion signal and an HA-tag sequence, was cloned into a lentiviral transfer vector, under the control of EF-1α promoter (Fig. 3A). Mouse SCs were transduced with resulting LVs to generate SC secreting BNb-PD1 (SC-BNbPD1) and SC secreting BNb-CSF1R (SC-BNbCSF1R) (Figure S5A). Western blot analysis of cell lysates and conditioned medium confirmed secretion of Nbs by engineered SCs (Fig. 3B, raw data in S7A). Dot blot assay further showed increasing concentrations of the SC released biparatopic Nbs in the conditioned media over time, in contrast to a single dose of Nbs in the media, which degraded over the course of three days (Figure S5B, raw data in S7C). To assess the functionality of SC-BNbPD1, tumour cell killing assays were performed using co-culture of tumour cells, T cells and SC-BNbPD1. Co-culture with SC-BNbPD1 resulted in a decreased viability of lung TC1 and melanoma UV2 tumour cells in comparison to the co-culture with control SC-GFP group, One-Way ANOVA (Fig. 3C and D). In parallel, splenocytes pre-activated with PMA/ion were incubated with conditioned medium from SC-BNbPD1. No significant changes in CD4+ or CD8+ T cell numbers were observed, but a significant increase in CD69-positive CD4+ and CD8+ T cells was detected, compared with controls, indicating enhanced activation of regulatory and cytotoxic T cell subsets, One-Way ANOVA, Tukey's multiple comparisons (Fig. 3E and F). To further these findings, tumour cells, activated splenocytes and SC-Nbs were co-cultured and additional T-cell phenotypes were analysed. In the presence of BNb-PD1, a significant reduction in the proportion of Treg population (CD4+ CD25+) and in LAG3+ expression within the CD4+ T cell component was observed, One-Way ANOVA, Tukey's multiple comparisons (Figure S5C). Similarly, analysis of CD8+ T cells revealed a significant decrease in the expression of CD25+, LAG3, and Tim3+ markers in the presence of BNbPD1, suggesting an overall more activated and less immunosuppressed T cell phenotype, One-Way ANOVA, Tukey's multiple comparisons (Figure S5C). To evaluate the functionality of SC-BNbCSF1R, BMDM were incubated with the conditioned medium from SC-BNbCSF1R. This resulted in a significant decrease in BMDM proliferation, Two-Way ANOVA (Fig. 3G). Next, we evaluated the expression of various surface markers on BMDM following incubation with SC-BNbCSF1R conditioned medium or to a commercial Ab against CSF-1R (AFS98, M2-Ab condition). Interestingly, BNb-CSF1R increased expression of markers associated with an anti-tumoural MΦ phenotype, such as MHCII, while reducing the cell surface expression of pro-tumoral markers, such as CD163, CSF-1R and PD-L1, One-Way ANOVA, Tukey's multiple comparisons (Fig. 3H and I). Taken together, these findings revealed that biparatopic Nbs secreted by engineered SCs are able to activate anti-tumour T cell response and inhibit MΦ proliferation *in vitro*.

### BNb-PD1 reduces tumour growth and BNb-CSF1R re-educates MΦ within the TME

**Fig. 4:**
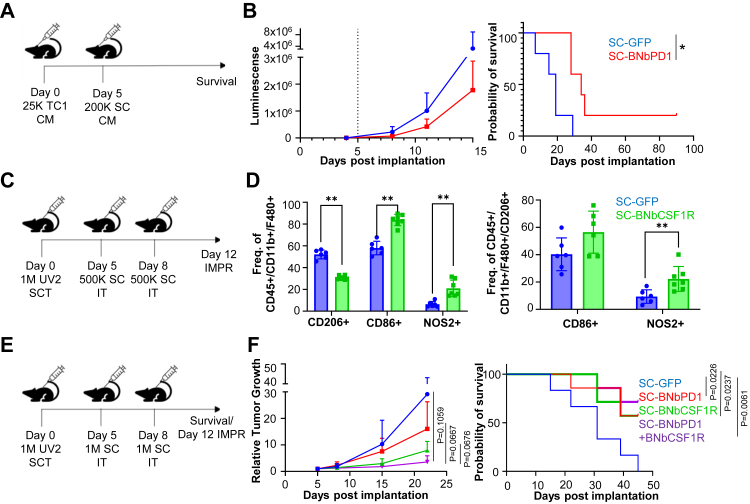
**In vivo functionality of SC secreting biparatopicNbs.** A) Schematic representation of the *in vivo* plan. Tumour cells and therapeutic SCs were implanted in the Cisterna Magna (CM). B) Tumour growth and survival of the mice treated (n = 5) with SC-BNbPD1. ∗, P = 0.0173 (Mantel–Cox test). C) Schematic representation of the *in vivo* experimental design. Tumours were implanted subcutaneously (SCT), followed by intratumoral (IT) SC implantation. D) percentage of CD206+, CD86+ and NOS2+ positive cells within the ΜΦ (CD45+/CD3-/F480+/CD11b+) population and expression of CD86+ or NOS2+ within the CD45+/CD3-/F480+/CD11b+/CD206+ population in presence SC-BNbCSF1R (n = 6 for SC-GFP, n = 7 for SC-BNbCSF1R), t-test, P < 0.05. E) Schematic representation of the treatment plan *in vivo* of SC-BNbPD1 and/or SC-BNbCSF1R treatment. F) tumour volumes and survival post SC-BNbPD1 and/or SC-BNbCSF1R treatment are presented (n = 6 for SC-GFP, n = 7 for all experimental). Statistical analysis of tumour volume and survival curves was with two-way ANOVA with Tukey's multiple comparisons test and Mantel–Cox test, respectively. Survival comparison between SC-GFP and SC-BNbPD1+BNbCSF1R was significant with the Bonferroni correction. (∗P < 0.05, ∗∗P < 0.01).

Next, we evaluated the functionality and therapeutic efficacy of SC-BNbCSF1R and SC-BNbPD1 *in vivo* across multiple tumour models. To determine the *in vivo* persistence of the Nb-based delivery platform, we first assessed whether Nbs secreted by engineered SCs remained detectable within tumours one week post-intratumoral injection, and compared this with intravenously administered commercial Abs targeting PD-1 and CSF-1R. ELISA studies indicated that SC-mediated delivery sustained detectable Nb levels within tumours for at least seven days, which was comparable to the persistence observed with systemically administered Abs (Figure S6A). Next, to assess the *in vivo* function and therapeutic efficacy of SC-BNbPD1, an intrathecal implantation model was employed to mimic leptomeningeal metastasis of lung cancer. Mice bearing TC1-GFP-Fluc leptomeningeal tumours were treated with SC-BNbPD1 or control SC-GFP. Tumour size was monitored using bioluminescence and mice were followed for survival (Fig. 4A). Reduced tumour volume and improved survival outcome were observed in mice treated with SC-BNbPD1 compared to mice treated with SC-GFP, with 20% of mice achieving long-term survival in the SC-BNbPD1 group, Mantel–Cox test (Fig. 4B). Next, we evaluated the effect of SC-BNbCSF1R on MΦ polarisation in subcutaneous melanoma UV2 tumours (Fig. 4C). Two intratumoral injections of SC-BNbCSF1R resulted in significantly lower percentage of CD206+ cells, a marker associated with an M2-like phenotype, within the tumour infiltrating MΦ population (CD45+CD3^−^CD11b + F4/80+ cells), compared with tumours treated with SC-GFP, t-test (Fig. 4D). Furthermore, tumours treated with SC-BNbCSF1R exhibited a higher percentage of CD86+ and NOS2+ markers reflecting an M1-like phenotype in the total TAMs population as well as TAMs expressing an M2 marker CD206, t-test (Fig. 4D). These results indicate that therapeutic SCs secreting biparatopic Nbs against PD-1 and CSF-1R are able to reprogramme the TME through PD-1 blockade, reducing tumour progression, and reversing TAM polarisation, respectively.

### Stem cell delivery of biparatopic Nbs against PD-1 and CSF-1R improves suppression of tumour growth

We hypothesised that blocking CSF1/CSF-1R and PD-1/PD-L1 would enable targeting both immunosuppressive TAMs and exhausted T cells, leading to increased activation of cytotoxic T cells and improved anti-tumour immune responses. To assess the impact of this dual targeting on immune populations within the TME, mice bearing subcutaneous UV2 melanoma tumours were treated by intratumoral injection of SC-BNbPD1 or SC-BNbCSF1R or combination of both SC-BNbPD1 and SC-BNbCSF1R, Two-Way ANOVA (Fig. 4E). Combined treatment of SC-BNbPD1 and SC-BNbCSF1R slowed tumour growth, compared to the control and the monotherapy of SC-BNbPD1 or SC-BNbCSF1R treatment, resulting in an increased survival in treated mice, Mantel–Cox (Fig. 4F). These findings indicate that blocking CSF1/CSF-1R and PD-1/PD-L1 axes potentiates anti-tumour immunity by targeting both immunosuppressive TAMs and exhausted T cells, leading to reduced tumour progression and increased survival outcomes.

### Intratumoral SC-BNbPD1/BNbCSF1R leads to TME immune reprogramming of tumours

**Fig. 5:**
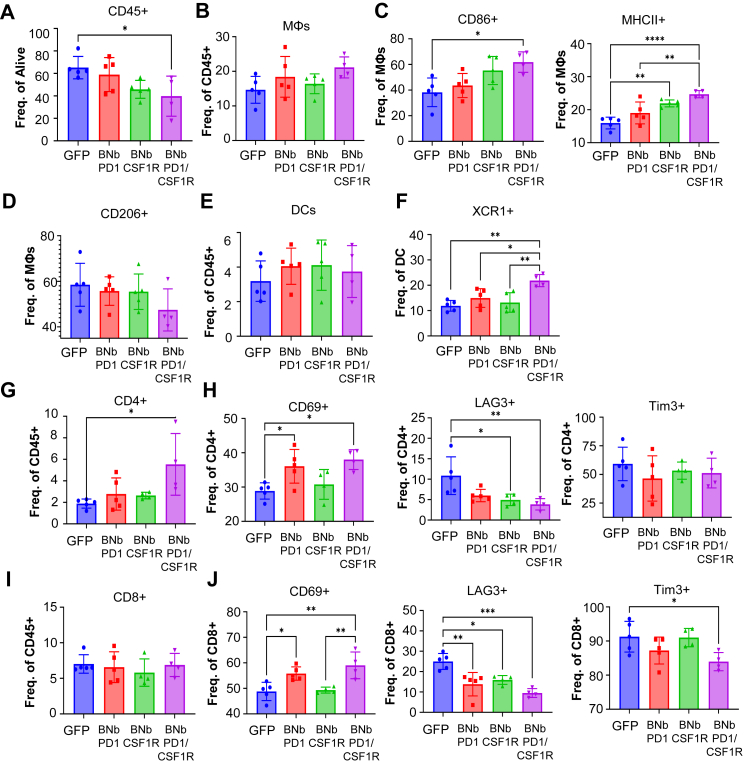
**Mechanism of the TME immune population activation through simultaneous treatment with SC-BNbPD1 and SC-BNbCSF1R.** A) Graph showing the infiltration of CD45+ cells in the tumours. B, C, D) Graph showing the MΦ population percentages in the within the tumour, and expression of MΦ polarisation markers CD86, MHCII and CD206. E) Graphs representing the percentage of DCs within the TME and the expression of XCR1 activation marker. F, G, H) Graphs showing the CD4+ T cells within the TME along with activation markers expressed on their surface. I, J) Same representations for the CD8+ T cell compartment. All the above results were analysed for significance using one-way ANOVA with Tukey's multiple comparisons test. For all the above n = 5 for GFP, BNbPD1 and BNbCSF1R, and n = 4 for BNbPD1+BNbCSF1R. (∗P < 0.05, ∗∗P < 0.01, ∗∗∗P < 0.001).

To elucidate the immunological effects of SC-NbPD1/NbCSF1R treatment, UV2 melanoma tumours were subjected to immune profiling of the TME. The combined SC-BNbPD1 and SC-BNbCSF1R treatment resulted in a reduction in the overall percentage of immune cells (CD45+) within the TME, One-Way ANOVA, Tukey's multiple comparisons (Fig. 5A). However, within the CD45+ population, the MΦ population (CD11c-F4/80+CD11b+) showed an upward trend relative to controls (Fig. 5B). Next, we analysed the expression of several activation markers on TAMs. Both CD86+ and MHCII+, markers associated with an anti-tumoural phenotype, were significantly upregulated in the SC-BNbPD1/SC-BNbCSF1R group compared to the control and monotherapy groups, One-Way ANOVA, Tukey's multiple comparisons (Fig. 5C). In contrast, M2 associated marker, CD206+ expression showed a downward trend (Fig. 5D), collectively indicating that combination treatment promoted TAM repolarization. Analysis of DC compartment (CD45+CD11c + F4/80-) revealed no significant changes in overall DC numbers across treatment groups. However, in the proportion of XCR1+ DCs was significantly increased in tumours treated with SC-BNbPD1/SC-BNbCSF1R compared to the control groups, suggesting enhanced DC activation of in the presence of both biparatopic Nbs, One-Way ANOVA, Tukey's multiple comparisons (Fig. 5E and F).

Within the T lymphocytes compartment (CD45+CD3+), the number of CD4+ T cells (CD45+CD3+CD4+) was increased in the SC-BNbPD1/SC-BNbCSF1R treatment group compared to the controls, One-Way ANOVA, Tukey's multiple comparisons (Fig. 5G). CD4+ T cells from this treatment group also expressed significantly higher CD69+ activation marker and exhibited lower levels of LAG3+ exhaustion marker (Fig. 5H), while the expression of Tim3 remained unchanged, One-Way ANOVA, Tukey's multiple comparisons (Fig. 5H). There were no differences observed in the overall frequency of CD8+ T cells between the treatment groups (Fig. 5I). However, within this population, CD69+ was significantly overexpressed in the combination group, alongside reduced proportions of LAG3+ and Tim3+ cells compared with controls, One-Way ANOVA, Tukey's multiple comparisons (Fig. 5J). Taken together, these results indicate that the combined SC- BNb-CSF1R/SC-BNb-PD1 is effective in reversing T cell exhaustion within the TME and contributing to suppression of tumour growth.

### Encapsulated SC-BNbPD1 and SC-BNbCSF1R therapy suppresses tumour growth and improves the survival probability following GBM tumour resection

**Fig. 6:**
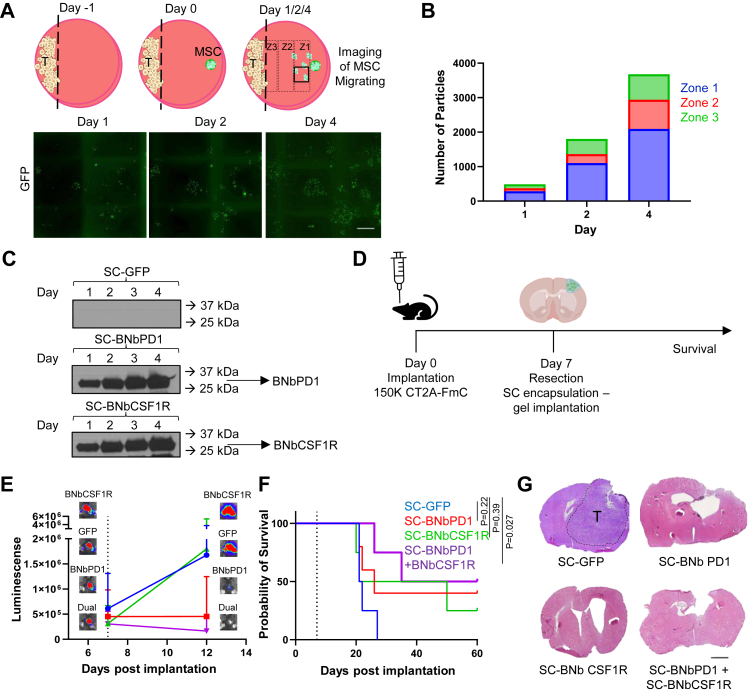
**Treatment with SC secreting BNbPD1 and/or BNbCSF1R in the resection cavity suppresses GBM growth.** A) Schematic and imaging showing the encapsulated SC migration out of the gel. B) Graph visualisation of the migrating SC, indicating their increased migration over four days. Dashed rectangles depict the migration zones and square on the scheme represents the area shown in the images below. C) Western Blot depicting the release of the biparatopic Nbs from SCs encapsulated in a gel over four days. D) Schematic showing the *in vivo* experiment plan. E, F, G) tumor volumes (E and F) and survival (G) post treatment with encapsulated SC-BNbPD1 and/or SC-BNbCSF1R in the resected cavity are presented, n = 4 (n = 5 for SC-BNbPD1). Dotted line in (F): day of tumor resection. One-way ANOVA analysis revealed no statistical significance in (E). Statistical analysis of survival in (F) used Mantel–Cox test (∗P < 0.05). H) Haematoxylin & Eosin staining of representative mice showing the tumor growth in mice treated with encapsulated SC-GFP and the complete regression in mice treated with SC-BNbPD1 and/or SC-BNbCSF1R. Scale Bar, 1500 μm applies to all images in A and G.

The current standard treatment for patients with GBM is maximal surgical resection, followed by temozolomide and radiation. As tumour resection is the first line of treatment in patients with aggressive GBM,^45^ we next tested the efficacy of SC-BNbPD1 and SC-BNbCSF1R in a highly immunosuppressive (CT2A) GBM surgical resection model. Given that the retention of therapeutic SCs at the resection site is key to the sustained delivery of therapeutic agents, we encapsulated SCs in pre-manufactured porous gel Celldex capsules, as previously described.^42^ We first confirmed the migration of the SC out of the gel capsule in a co-culture with CT2A-FmC GBM cells (Fig. 6A). Encapsulated SC migrated out of the gel, which increased over the course of four days, starting from the gel side, heading towards the tumour site on the plate (Fig. 6B). Western blot analysis of the supernatant from engineered SCs encapsulated in gels over four days revealed the release of BNbPD1 and BNbCSF1R during this period, as evidenced by their increasing concentration in the supernatant, indicating the Nbs capacity to escape from the gel (Fig. 6C, Figure S5B, raw data in S7B). Next, we tested the encapsulated SC-BNbPD1/BNbCSF1R in mice bearing GBMs. Mice bearing highly malignant immunosuppressive brain tumours CT2A-FmC were resected and treated with a single dose of encapsulated SC-BNbPD1/BNbCSF1R (Fig. 6D). This treatment suppressed tumour growth compared to the SC-BNbCSF1R and control SC-GFP groups (Fig. 6E) and resulted in increased survival probability compared to the control group, Mantel–Cox test (Fig. 6F). At the end of the survival study, at 60 days post tumour implantation, the brains of the remaining mice were sectioned and stained with Haematoxylin and Eosin, verifying the tumour was completely eradicated in the survivors following the treatment with Nbs (Fig. 6G). Additionally, potential toxicities of SCs were assessed by examining the pathology of major organs, such as the spleen, kidney, brain and liver. No notable differences in pathology were observed, indicating that the therapeutic SCs did not cause toxicities in mice (Figure S6B). In conclusion, our findings demonstrate that the combination of encapsulated SC-BNbPD1 and SC-BNbCSF1R effectively targets the immunosuppressive environment of GBM following surgical resection, underscoring the potential of this approach.

## Discussion

In this study, we generated Nbs targeting CSF-1R and PD-1 in llamas and created their biparatopic variants to re-educate TAMs and overcome T cell exhaustion in the TME. We subsequently developed a platform of allogenic off-the-shelf SCs releasing biparatopic PD-1 and CSF-1R Nbs and evaluated its efficacy across multiple mouse tumour models. Our findings show that syngeneic SC mediated delivery of biparatopic Nbs resulted in reduced tumour growth via immune activation of T cells and DCs and re-education of MΦ phenotypes.

Previous studies have shown that increased expression of CSF-1R within the TME is associated with increased expression of PD-1 and PD-L1 and increased M2-like TAMs, collectively correlating with poor outcome.^46^, ^47^, ^48^ These findings were confirmed by our TCGA analysis. Notably, simultaneous overexpression of PD-1 and CSF-1R was also linked to reduction in activated DC populations. CSF-1R overexpression on TAMs has been observed in various tumour types,^49^ contributing to tumour progression by promoting immunosuppression, tissue remodelling, angiogenesis and tumour cell migration.^37^^,^^50^, ^51^, ^52^ In parallel, PD-L1 is expressed on the surface of multiple cell types within TME, including pro-tumoral TAMs and tumour cells.^53^^,^^54^ Elevated PDL-1 expression on TAMs indirectly suppresses the anti-tumour immune response by inhibiting T-cell activation and promoting T-cell exhaustion, thereby further contributing to T cell exhaustion, ultimately promoting tumour growth.^53^^,^^55^

We developed Nbs targeting PD-1 and CSF-1R to inhibit CSF1/CSF-1R and PD-L1/PD-1 axes respectively. After generating several monovalent Nbs clones and testing their binding affinity against each target, the two highest affinity clones were then used to develop biparatopic Nbs. Competition assays along with predictive protein docking modelling verified that each Nb of the biparatopic Nbs binds to different epitopes on PD-1 and CSF-1R, respectively. Notably, NbPD150 exhibited a slightly better binding capacity than BNb-PD1. Despite this, we found that BNb-PD1 was more effective at blocking the PD-1/PD-L1 pathway and mediated higher tumour cell killing by T cells *in vitro*. This apparent discrepancy between binding affinity and functional efficacy is likely explained by the increased spatial occupancy of PD-1 and the receptor conformational changes induced by biparatopic Nbs engagement, as previously described^6^^,^^8^ and supported by our predictive modelling studies (see Fig. 2G and H). Consistent with this, BNb-CSF1R also showed superior functional efficacy, inhibiting BMDM proliferation. This observation is consistent with previous studies describing the central role of CSF-1 signalling in MΦ proliferation.^56^^,^^57^

As SC delivery enables continuous secretion of therapeutics directly within the TME, thereby overcoming the limitations associated with systemic delivery and rapid clearance of Nbs,^58^^,^^59^ we engineered SCs to secrete BNb-CSF1R or BNb-PD1. Co-culture of tumour cells and activated T cells in the presence of SC-BNbPD1 led to nearly 50% tumour cell killing in both TC1 and UV2 cell lines. PD-1 blockade in activated splenocytes led to increased expression of CD69 on the surface of T cells, while CD4+ and CD8+ T cell numbers remained unchanged, indicating increased T cell activation.^60^ Additionally, Tregs and Tim3 and LAG3 exhaustion markers significantly decreased in the presence of BNbPD1. Our findings are consistent with previous reports that PD-1/PD-L1 blockade reverses T cell exhaustion via the removal of the inhibitory PD-L1/PD-1 signal, leading to increased expression of activation markers and cytokine production.^61^^,^^62^ SC-derived BNb-CSF1R lowered the MΦ proliferation rate over a course of three days, mimicking the conditions in which CSF1 is absent or CSF-1R is neutralised with blocking Abs. We also examined the expression of various M1 like markers (CD86 and MHCII) and M2-like markers (CD163, PD-L1, and CSF-1R) in response to SC-secreted BNb-CSF1R.^63^ Consistent with previous studies, showing that CSF1 drives MΦ polarisation towards a pro-tumoral phenotype, and blockade of CSF1/CSF-1R axis promotes MΦ re-education to an M1-like phenotype,^17^^,^^18^^,^^64^^,^^65^ our results show that SC-secreted BNb-CSF1R to increase the expression of M1-like markers and reduce the expression of M2-like markers in BMDM.

*In vivo*, SC-secreted BNb-PD1 in the TME of lung to brain metastasis slowed tumour growth and increased animal survival. In UV2 melanoma, SC-secreted BNb-CSF1R was sufficient to increase the TAMs exhibiting M1-like markers. Interestingly, within the population of M2-like TAMs, the M1-like markers were increased in the BNb-CSF1R treated group compared to the control group. While the antitumour effects of the combination therapy improved survival over monotherapy without reaching statistical significance, these findings are valuable for understanding the therapeutic landscape. More specifically, co-treatment with SC-BNbPD1 and SC-BNbCSF1R in both melanoma and GBM models was most potent in slowing down the tumour growth, indicating that the presence of both Nbs in the TME had the most beneficial impact. Moreover, local production of BNb-CSF1R and BNb-PD1 in the TME conferred a significant survival benefit over the control group, suggesting that targeting a single immunosuppressive pathway might not be sufficient to achieve durable survival benefits. These data are supported by immune profiling studies, where we observed significant landscape re-education when both BNbPD1 and BNbCSF1R were present in TME. Importantly, SC-BNb-PD1 treatment significantly increased CD69 and decreased LAG3+, cell surface markers of activation and exhaustion, respectively^66^ in both CD4+ and CD8+ T cell populations, while SC-BNbPD1 and SC-BNbCSF1R significantly reduced Tim3 levels in CD8+ T cells. Similar to PD-1, prolonged LAG3 signalling leads to T cell exhaustion, and some tumours are characterised by high LAG3 expression within the TME.^66^, ^67^, ^68^ Previous studies have shown a cross-talk between PD-1/PD-L1 and LAG3 signalling pathways and therefore, PD-1/PD-L1 blockade could disrupt the pathways that regulate LAG3 expression, leading to decreased expression of LAG3.^67^^,^^69^ Interestingly, exposure to both BNb-PD1 and BNb-CSF1R led to a significant increase of CD4+ T cells, indicative of higher T cell infiltration driven by SC-BNbPD1-mediated PD-1 blockade.^70^^,^^71^ Moreover, our findings indicate that SC release of BNb-CSF1R within the TME led to further DC activation which is crucial for antigen presentation and stimulation of CD4+ T cell responses.^72^ Both MΦs and DCs compartments exhibited a significant increase in pro-inflammatory activation markers when tumours were treated with SC-BNbPD1 and SC-BNbCSF1R.

Previous studies have shown that CSF-1R blockade leads to TAM reeducation in solid tumours.^16^^,^^18^^,^^48^^,^^73^ Anti-inflammatory phenotypes in TAMs usually exhibit increased PD-1 and PD-L1 on the surface, inhibiting their phagocytosis functions and suppressing DC anti-tumour activity. Collectively, SC secretion of BNb-PD1 and BNb-CSF1R within the TME leads to T cell reactivation, MΦ repolarization, and DC activation through direct and interactive effects. Although we did not investigate in the current work, signal regulatory protein alpha (SIRPa) on TAMs and CD47 on tumour cells form an axis that functions as a “don't eat me” signal, which inhibits MΦ-mediated phagocytosis and enables tumour cells to evade immune clearance, and can potentially lead to further T cell exhaustion.^74^ SIRPa is frequently overexpressed in pro-tumoral TAMs across various cancers, contributing to immune evasion and sustaining a suppressive TME (73). Previous studies have demonstrated that targeting this axis can modulate TAM activity, promoting a shift towards a more inflammatory and antitumour MΦ phenotype, and promoting phagocytosis.^74^, ^75^, ^76^ While blockade of CSF-1R can reprogramme TAMs within the TME, combining our BNbCSF1R with an agent blocking CD47-SIRPa could synergistically reprogramme TAMs and bolster antitumour immune responses and tumour cell phagocytosis further.

Previous studies have investigated the combined blockade of PD1/PD-L1 and CSF1/CSF-1R in cancer models. A study investigated the combination of PD-1 and CSF-1R inhibition in pancreatic cancer, in which CSF1R and PD1 inhibitors were administered systemically.^48^ The dual blockade resulted in a reduction of tumour growth and increased infiltration of T cells in the TME, while enhancing the efficacy of chemotherapy and radiation.^48^ Similarly, a recent study in colorectal cancer demonstrated the benefit of CSF1R blockade in improving the response to PD-1 blockade Ab.^77^ The use of engineered SCs to locally release BNb-PD1 and BNb-CSF1R highlighted the potential of this approach in reducing tumour growth through immune activation of T cells and DCs and re-education of MΦs, by avoiding off target effects and systemic toxicity. We have previously shown that resection of the GBM tumour in mice mimics the first line of the treatment regimen in patient and results in the influx of activated T cells and decreases immune suppression.^35^ Moreover, we have encapsulated engineered SC in biocompatible HA based gels^35^^,^^78^ and gelatin-based gels^42^ and shown their efficacy in mouse tumour models of GBM resection. This strategy allows retention of engineered SC at the tumour site and preventing them from washing-out.^42^ Our gelatin-based encapsulation approach enables the pre-manufacturing of therapeutic capsules and ensures batch-to-batch consistency. Treatment of resected immunosuppressive CT2A tumours with encapsulated SC-BNbPD1 and SC-BNbCSF1R led to improved survival. These data substantiate the translational rationale of using locally released BNb-PD1 and BNb-CSF1R at the time of tumour resection to address the residual tumour cells. Further investigations are needed to elucidate the underlying mechanisms and explore the full therapeutic potential of this approach.

It is essential to acknowledge that our study is using SCs primarily used as vehicles to achieve successful sustained secretion of biparatopic Nbs in the TME. While our findings utilise immunosuppressive tumour models in an immunocompetent mouse setting and offer a good insight into the immune effect of this delivery approach, further optimisation in humanised mice will be required before translating to human settings. To date, several SC-based therapies have been tested in clinical trials for various cancer types.^79^ These therapies typically involve the use of SCs to modulate the immune response or deliver targeted therapies directly to tumours. Moreover, SC based therapies are being evaluated in solid tumours and haematological malignancies, with several trials in Phase I and II, assessing the use of SCs for delivering anti-cancer agents or modulating the immune microenvironment.^79^^,^^80^ Data from limited early studies suggest that SCs help enhance the effectiveness of other therapies, but data on long-term efficacy is still emerging. Continued research and technological refinement will therefore be essential to drive translational advancement towards clinical implementation.

Overall, this study highlights the successful development and characterisation of Nbs targeting PD-1 and CSF-1R, as well as the enhanced functionality of biparatopic formats. The use of engineered SCs secreting these Nbs demonstrates potential as a therapeutic approach for modulating the immune response and promoting anti-tumour effects in different tumour types and may offer a strategy to sensitise cancers resistant to the existing immunotherapies.

## Contributors

Conception: K. Shah, Experimental design: K. Shah, J. Gettemans I. Vogiatzi; Development of methodology: K. Shah, J. Gettemans, I. Vogiatzi, L. Moreno Lama, M. Rashidian; Acquisition of data: I. Vogiatzi, A. Lehmann, O. Zwaenepoel, N. Kanaya, C. Liu, Y. Kajiwara, F. Rossignoli, A. Farrid, U.J. Lee; Analysis and interpretation of data (e.g., statistical analysis, biostatistics, computational analysis):.Vogiatzi, A. Lehmann, K. Shah, J. Gettemans; Writing, review, and/or revision of the manuscript: I. Vogiatzi, A. Lehmann, L. Moreno Lama, K. Shah, J. Gettemans, H. Wakimoto; Administrative, technical, or material support (i.e., reporting or organising data, constructing databases): J. Gettemans I. Vogiatzi; K. Shah; Study supervision: K. Shah, J. Gettemans; Authors that have accessed and verified data presented: IV, FR, HW, KS; All authors have read and approved the manuscript.

## Data sharing statement

All raw data are available and will be shared upon request.

## Declaration of interests

K.S. owns equity in and is a member of the Board of Directors of AMASA Therapeutics, a company developing stem cell-based therapies for cancer. K.S.’s interests were reviewed and are managed by Brigham and Women's Hospital and Partners HealthCare in accordance with their conflict-of-interest policies. J.G. owns Gulliver Biomed bv. The other authors declare that they have no competing interests.

## Appendix B Supplementary data

Supplementary Tables

Supplementary Figures Caption

Uncropped Western Blots
