# Supplementary figures and images for: A nanobody-stem cell platform targeting innate and adaptive immune axis in the tumour microenvironment
Source: eBioMedicine. 2026 Jan 17;124:106122. doi: 10.1016/j.ebiom.2026.106122 (PMC12853783; doi:10.1016/j.ebiom.2026.106122)

## Supplementary Figure 1

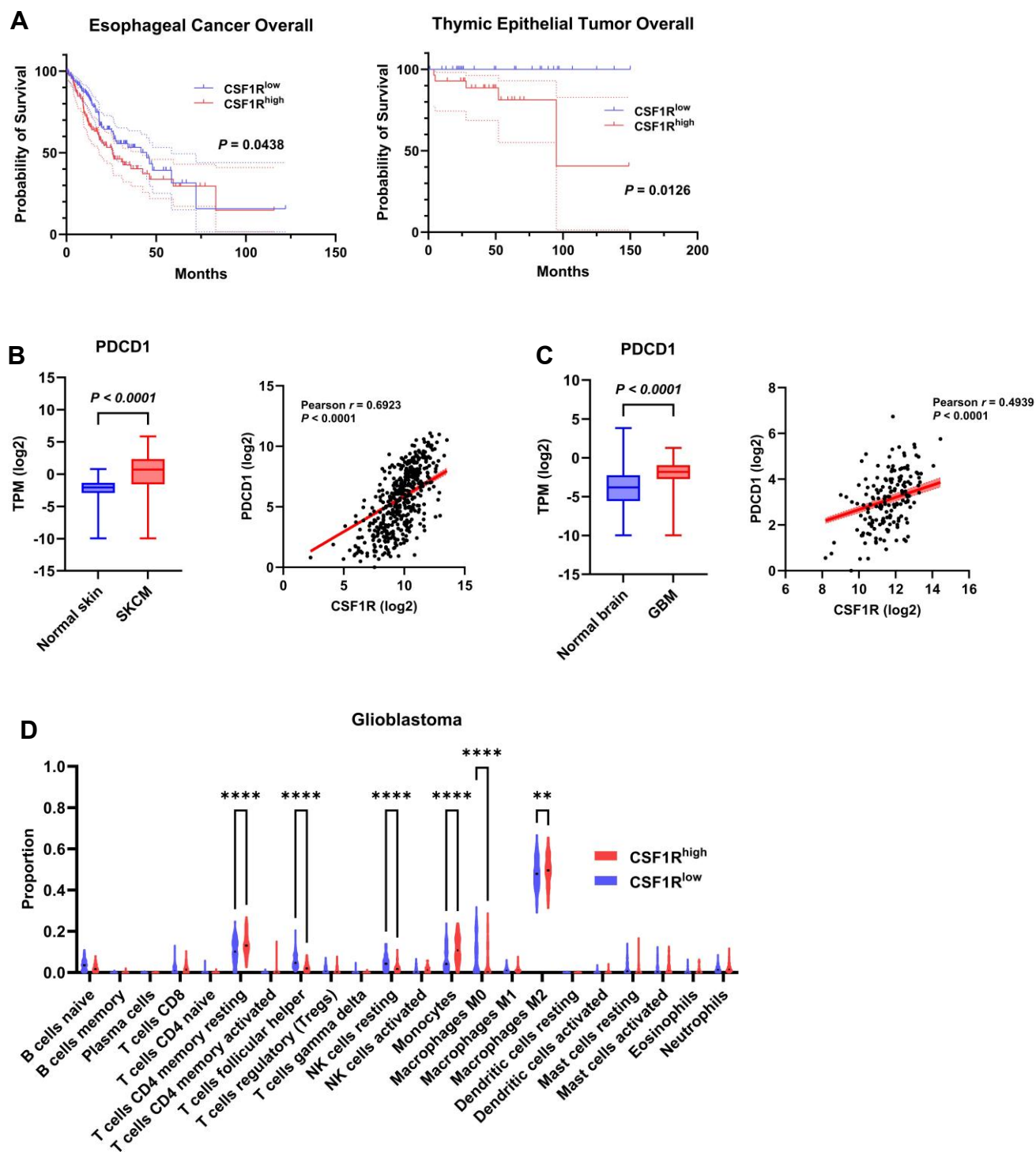

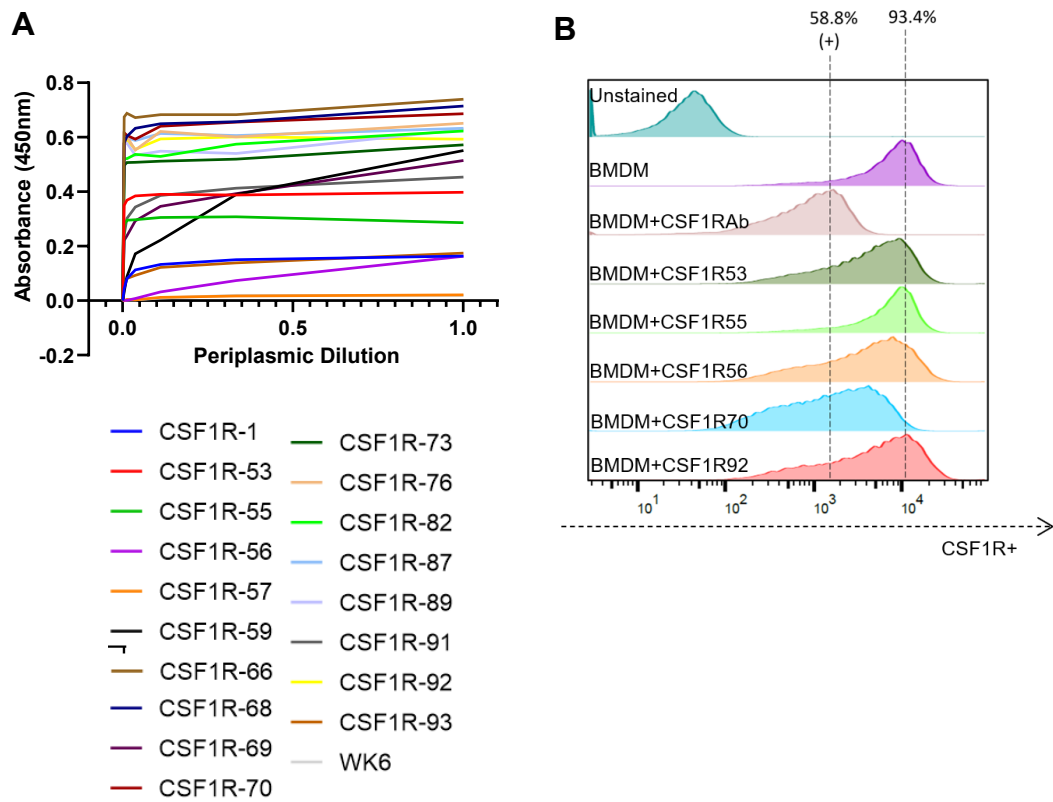

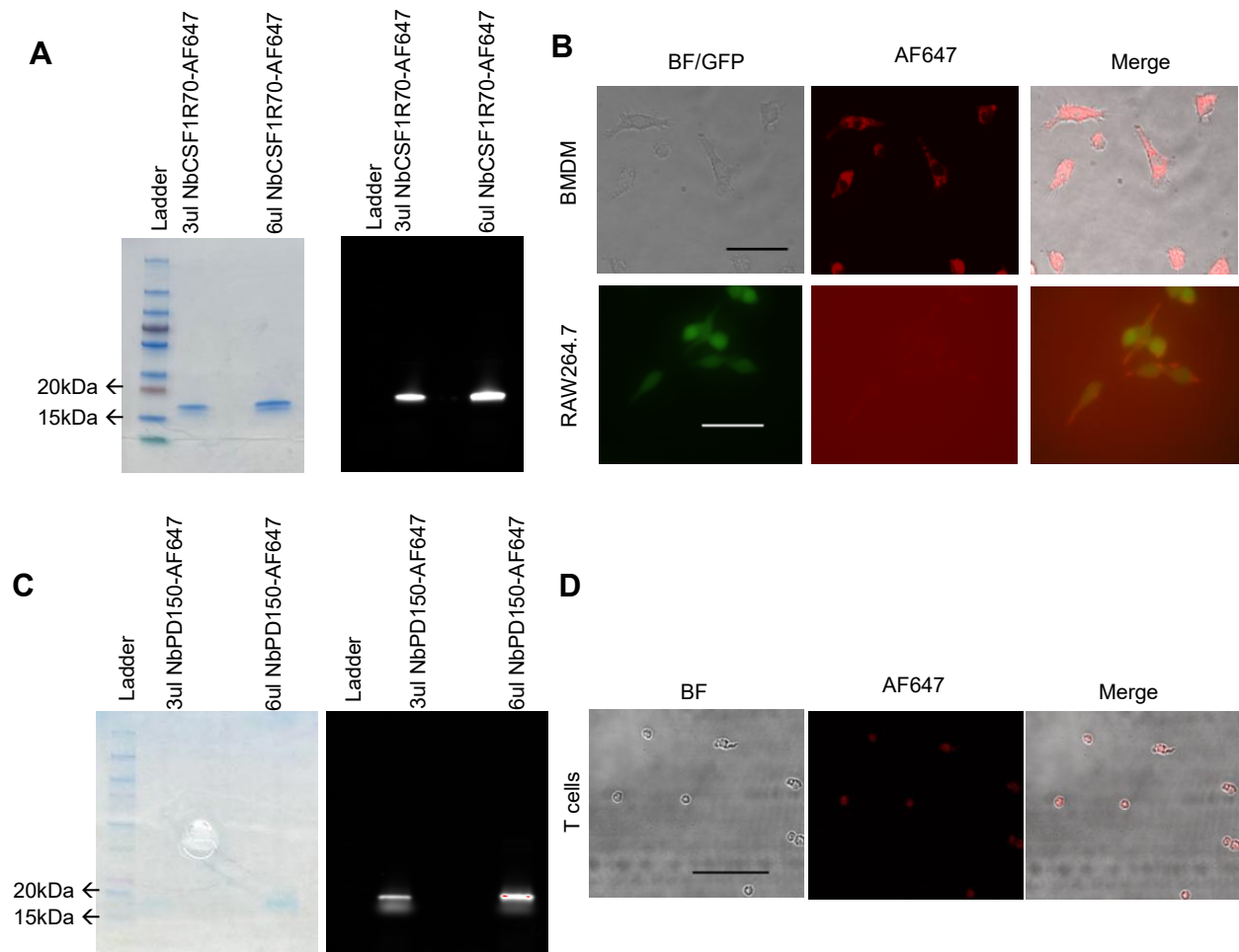

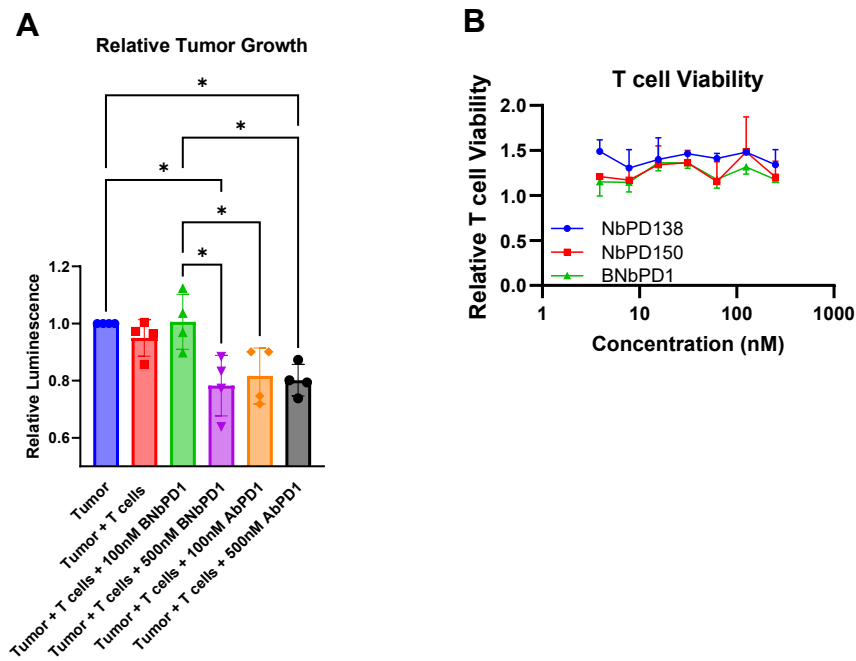

**A**

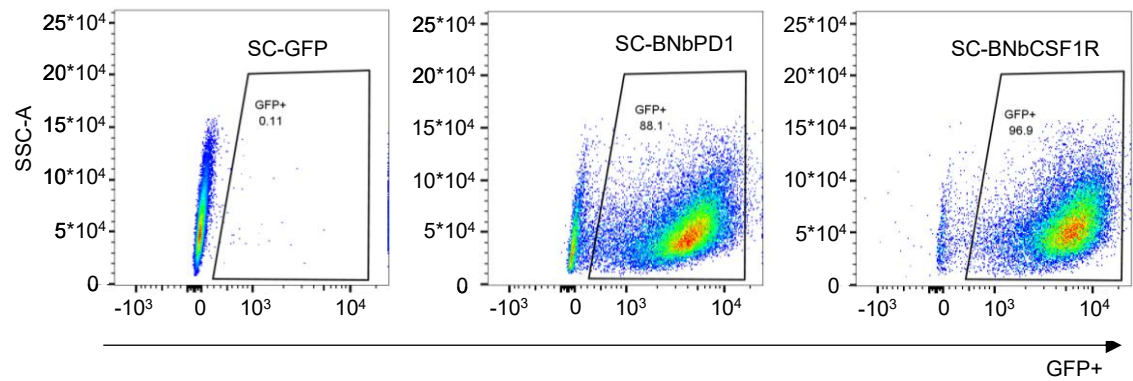

**B**

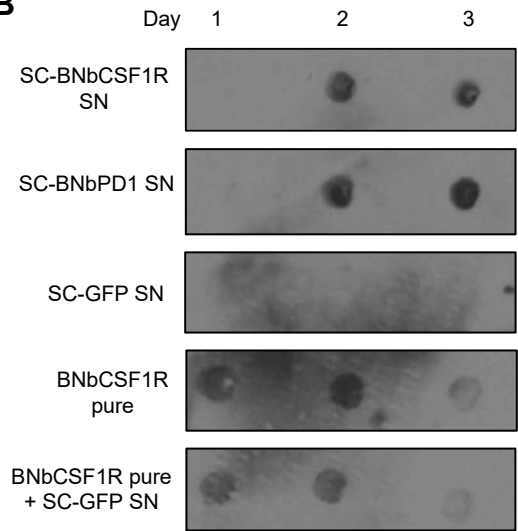

**C**

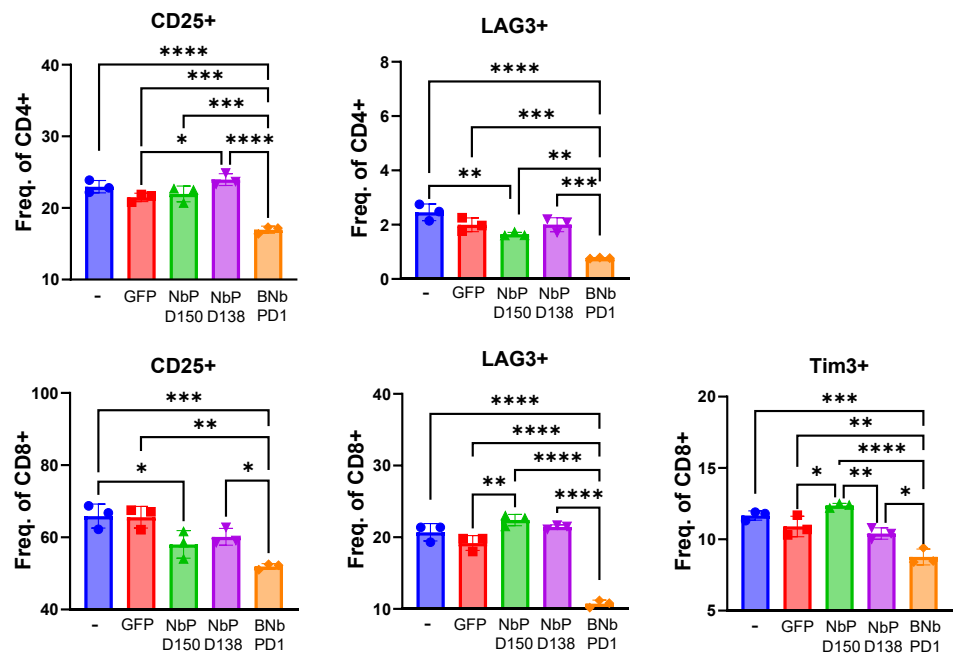

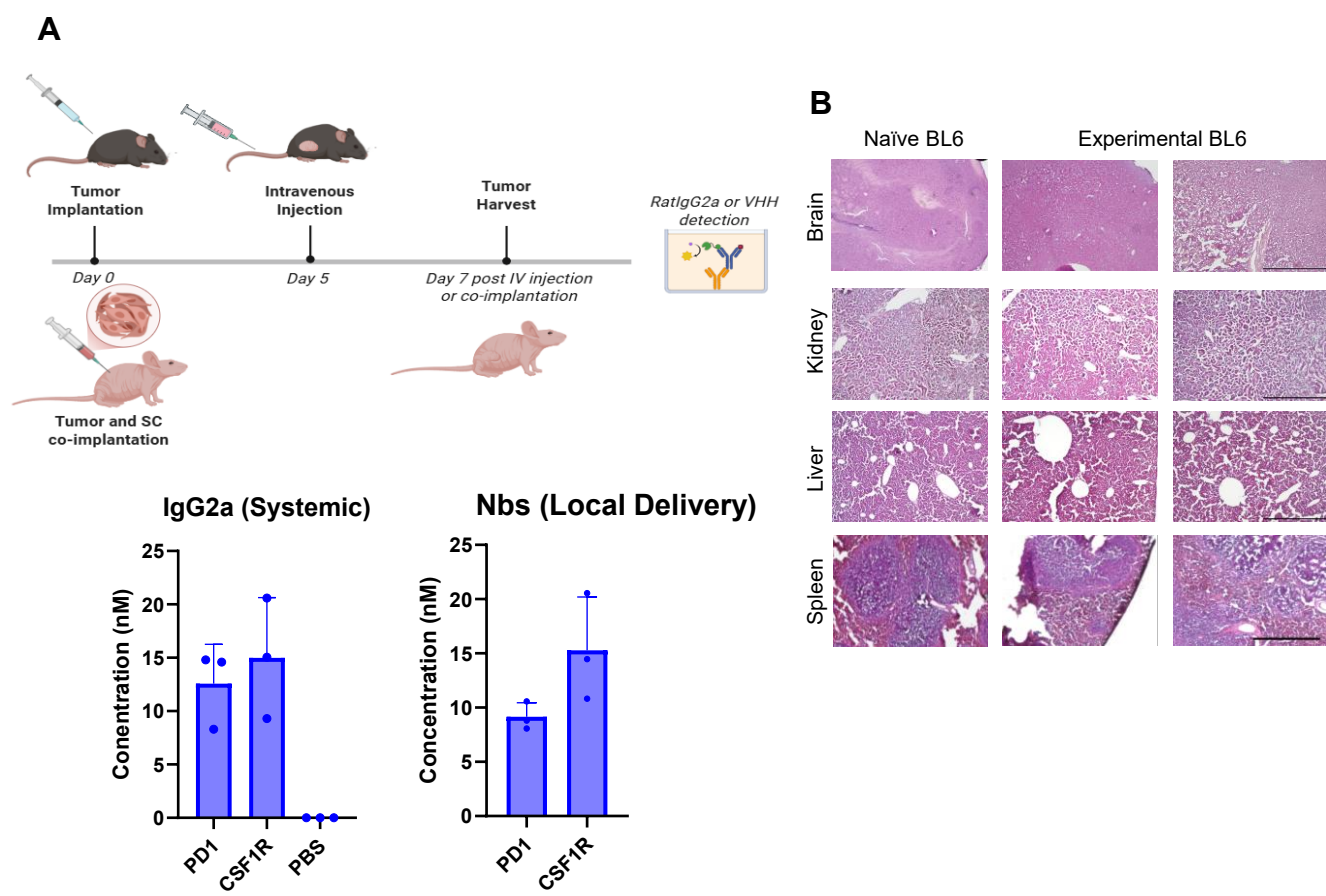

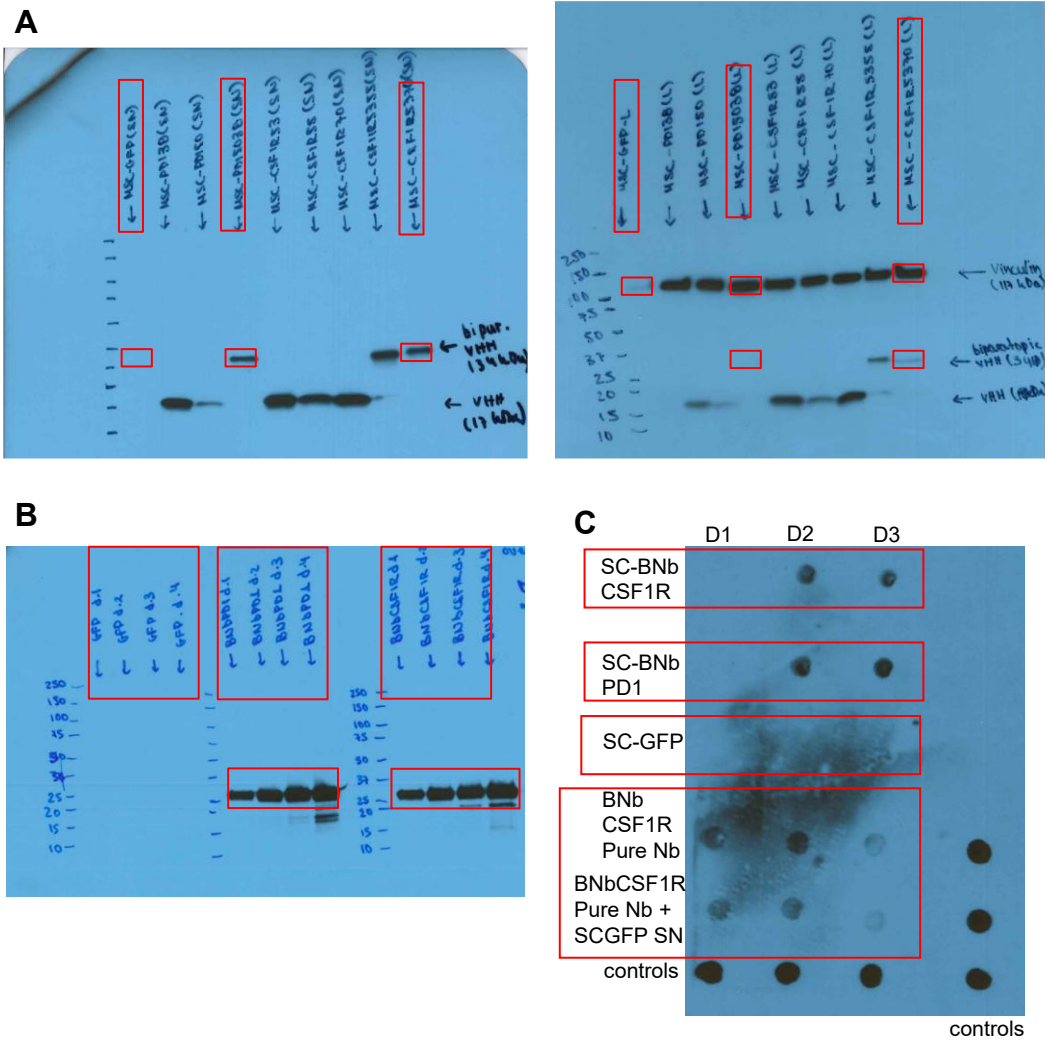

Supplement: Supplementary Figures [file mmc1.pdf]
