# Supplementary material for: A nanobody-stem cell platform targeting innate and adaptive immune axis in the tumour microenvironment
Source: eBioMedicine. 2026 Jan 17;124:106122. doi: 10.1016/j.ebiom.2026.106122 (PMC12853783; doi:10.1016/j.ebiom.2026.106122)
Supplement: Supplementary Tables [file mmc2.docx]

**Supplementary Methods**

**TCGA analysis:** RNA-seq data were analysed using CIBERSORTx (<https://cibersortx.stanford.edu/>), cBioPortal (<https://cbioportal.org>) and UCSC Xena (https://xena.ucsc.edu/), employing predefined immune cell marker gene sets representing specific immune cell types. Processed RNA-seq data were uploaded to the CIBERSORTx web platform, where linear regression and machine learning algorithms calculated the relative proportions of various immune cell types present within the samples. Immune cell analysis was conducted specifically for PanCancer (n=4989), GBM (n=166), lung adenocarcinoma (n=506), skin cutaneous melanoma (SKCM) (n=472), oesophageal Cancer (n=295) and Thymic Epithelial Cancer (n=58). Survival data was divided into quartiles. The proportions of immune cells between the two groups were evaluated for statistical significance using a t-test based on median mRNA expression levels, with a p-value threshold set at 0·05. PD1 and CSF1R expression were categorized as high expression when both mRNA levels were above the median, and low expression when both were below the median. Extracted data were entered into GraphPad Prism 9 software for generating graphs and performing statistical analyses.

**Bone marrow cell isolation and culture from mice.** Bone marrow progenitor cells were isolated from naïve C57BL/6 mice using femurs and tibiae. Femurs and tibiae were flushed with 10mL cold sterile 1X PBS using a 26G needle and passed through a 70 mm filter (Cell Treat). Cells were adjusted to a concentration of ~4 × 10^6^/ml in macrophage complete medium (DMEM/F12 medium, 20% (v/v) L-929 conditioned medium, 1% P/S and 10mM GlutaMAX). For culture, 4 × 10^6^ cells per sterile petri dish were diluted in 9 ml macrophage complete medium (total volume of10 ml). Cells were incubated at 37°C, in a 5% CO_2_ incubator for seven days, with an additional 5ml of medium added per plate on day three. The differentiated macrophages were validated with flow cytometry for CD45+/CD11b+/F4-80+ expression and maintained in culture for maximum of 14 days post- differentiation.

**T cell isolation and T cell activation*.*** Splenocytes were isolated from female C57BL/6 mice aged 8-10 weeks, and T cells were separated using the EasySep™ Mouse T cell Isolation kit (STEMCELL^TM^ Technologies #19851). Following isolation, T cells were cultured in X-VIVO 15 medium (Lonza,04-418Q) containing 10% FBS and 50Units/ml IL2 recombinant protein. T cell activation was performed by incubating cells with PMA/ionomycin for 48-72hrs. Splenocytes were cultured and activated using the same protocol. Cells were isolated from individual spleens for each experiment to minimise variability between the biological replicates. All experiments were performed in triplicates.

**Binding Capacity of the Nbs.** The binding capacity and the affinities of the Nbs were evaluated with Periplasmic ELISA and ELISA with purified Nbs. For the periplasmic ELISA, 96 well plates (Nunc MaxiSorp™ flat-bottom, 44240411) were coated with 1μg/μl of purified murine CSF-1R (Sino Biological 50059-M08H) or PD-1 protein (Sino Biological, 50124-M08H) and incubated ON at 4^o^C. The following day, plates were washed three times with 1X PBS containing 0·05% Tween (Bio-Rad, 1706531) and blocked with 1X PBS containing 0·05% Tween and 4% powder milk (Bio-Rad, 1706404XTU) for one hour while shaking in 4^o^C. After discarding the blocking buffer, plates were incubated for one hour at 4^o^C, shaking with periplasmic titrations prepared in 1X PBS. periplasm was collected from the liquid cultures as described above. Following incubation, plates were washed three times with the same wash buffer, and the Nbs binding was detected with Rabbit-anti-HA tag C29F4 (Cell Signaling Technologies, 3724), followed by Goat-anti-Rabbit IgG HRP (Abcam, 97051). The HRP Signal intensity was developed with 50ul/well of 1 Step TMB-ELISA substrate (Thermo Fisher, 34028) and reaction was stopped with 50ul/well of 0·2M H_2_SO_4_. Signal intensity was measured at OD_450_. To determine the affinities of the Nbs, the same protocol was followed, using purified Nbs for titrations. All binding assays were performed in duplicate.

**Protein-Protein Binding Analysis.** The sequence and structure of PD-1 were modelled on the crystal structure (PDB ID: 3BIK). The binding poses of NbPD138 and Nb-PD150 with PD-1 were predicted using Alphafold 3(1). The BNbPD1 fusion protein was also modelled using Alphafold 3. Similarly, the binding poses of Nb-CSF1R53 and NbCSF1R70 with CSF1R were generated using Alphafold 3. and the BNbCSF1R fusion protein was predicted in the same way. Protein-protein binding poses were visualized by Chimera X software(2). The H1, H2, and H3 complementarity-determining regions (CDRs) were identified from the predicted structures. The optimal predictions were selected based on the orientation of the VHH CDRs ( H1, H2, and H3) relative to the antigen. Epitope sites were predicted by analysing the contact residues between the PD-1 or CSF-1R and the VHH’ H1, H2, and H3.

**Receptor occupancy.** BMDM were incubated with Nbs against CSF-1R or with unconjugated AFS98 CSF1RAb for 2 hours. Following incubation, BMDMs were stained with AFS98 CSF1RAb-AF647 and flow cytometry was performed to detect AF647 positive cells. Fewer AF647 positive cells indicate higher receptor occupancy.

**Competition assay.** Several dilutions of unconjugated Nbs were incubated with BMDM or T cells for 30 min followed by the sequential addition of fixed concentrations of conjugated Nbs. This allowed Nbs to bind to their respective epitopes in a stepwise manner. Results were assessed using flow cytometry, with n=2.

**DNA Cloning.** DNA sequences encoding the monovalent and the biparatopic Nbs were synthetized and cloned into third-generation lentiviral plasmid backbones under the control of EF1-α promoter, including a GFP marker for selection of transduced cells. Nb sequences also included a His Tag or HA tag for detection or purification of Nbs in downstream assays. For Nbs used in sortase mediated conjugation with fluorescent dyes, a sortase recognition tag was cloned into the pMECs vector.

**Sortase mediated conjugation of Nbs with Alexa Fluor647.** The Penta mutant sortase A was utilized for all experimental procedures. The reaction mixture contained 50 mM Tris-HCl (pH 7·5), 150 mM NaCl, 10 mM CaCl_2_, 2−10 μM sortase, and >5 mM triglycine (Gly3) and were incubated for 1 hour at 4°C. Reactions were quenched by with 25 mM EDTA, and proteins were purified using size exclusion chromatography.

To immobilize sortase on beads, dried NHS-activated agarose resin (Thermo) was employed to covalently bind with primary amines. Approximately 20 mg of sortase in 3 mL of HEPES buffer (50 mM, pH 7·5) was mixed with 150 mg of resin in a 15 mL tube and incubated overnight at 4 °C on a rotator. The tube was centrifuged at 1000g for 1 min. and beads were washed twice with 10 mL of HEPES buffer (50 mM, pH 7·5), and incubated in quenching buffer (1 M Tris, pH 7·4) for 30 minutes at room temperature. Following final wash, sortase-immobilized beads were preserved upright at 4 °C in HEPES buffer (50 mM, pH 7·5), ensuring at least 0·5 mL of buffer was above the resin.

**Western Blot Analysis*.*** 1x10^6^ cells were seeded and incubated OPTIMEM medium for 48 hours. Subsequently, the supernatants were collected, and the cell lysates were prepared using NP40 Lysis buffer. Protein concentrations were measured using Bradford Protein Assay and 30ug of protein per sample were loaded On a 4-20% gel (Biorad, 4561093) for electrophoresis, followed by protein transfer on nitrocellulose membrane (Biorad, 1620115). Proteins were detected with Rabbit anti-HA Tag (Cell Signaling Technology, C29F4) and Anti-Rabbit IgG -HRP (Cell Signaling Technology, 7074S). Membranes were developed using Super Signal West Pico Plus (Thermo Fisher Scientific, 34577)

**Cell viability assays*.*** Co-culture viability assays using T cells, TC1-GFP-Fluc or UV2-GFP-Fluc tumor cells and SC-GFP or SC secreting Nbs were performed with luciferase activity measured after two days. BMDM cells were incubated with monovalent or bivalent Nbs against CSF1R up to three days, in the presence or absence of CSF1 and/or an anti-CSF1R Ab (AFS98) antibody. T cell viability assays with varying Nb concentrations were performed over 3 days. Viability and growth were measured using Cell Titer Glo.

**Macrophage Polarization.** BMDM cells were stimulated for 24 hours with 200 ng/ml LPS (Sigma, L2630-10MG) for M1 like polarization or 10ng/ml IL-4 (Abcam, 9729) for M2 like polarization. The degree of polarization was determined by flow cytometry, (n=3)

**Gel encapsulation: A** 20 wt% GelMA solution was prepared by dissolving lyophilized GelMA in PBS under constant stirring at room temperature or 37 °C. A 10 wt% dextran solution (Mw = 2000 kDa, Aladdin) and 4 wt% lithium phenyl-2,4,6-trimethylbenzoylphosphinate (LAP, Advanced BioMatrix) solution was also prepared in PBS. Gels were formulated by to final concentration of 4·0 wt% dextran,10 wt% GelMA, 0·2 wt% of LAP, mixed with a PBS cell suspension. The capsules were solidified with 60 sec of UV light exposure. This method is extensively described by Van Schaik., et al.(3)

***In vitro* migration assays.** Mouse CT2A-FmC GBM cells were seeded on one side of the culture plates and incubated for 24 hours. SCs were then encapsulated within a gel matrix and wells were then imaged on days 1, 2, and 4 using a Nikon TI Eclipse microscope. For cell migration analysis , each well was delineated into three distinct zones: Zone 1 (Z1), located adjacent to the encapsulated gel; Zone 2 (Z2), situated between Z1 and Zone 3 (Z3); and Zone 3 (Z3), located near the tumor cells.

**Hematoxylin and Eosin Staining**: Brain, Liver, Spleen, and Kidney were sectioned (12 µm) using a cryostat, thawed and hydrated in PBS. Sections were then placed in undiluted Hematoxylin for 30 s, followed by five consecutive washes in dH2O, then treated with 70% Ethanol. Sections were stained with undiluted osin staining for 25 s followed by sequential washes in in 70% , 95% ( and 100% ethanol (two washes each, 2 min per wash) and a final 5 min. incubation in Xylene. Sections were then mounted and imaged.

**Flow cytometry and immune profiling analysis*.*** A subcutaneous melanoma model was established by implanting UV2 cells (1x10^6^ cells/mouse) into C57BL/6 mice (6 to 8 weeks). On day 5 and 8, therapeutic SC-BNbPD1 and/or SC-BNbCSF1R (1*10^6^/mouse) were administered intratumorally . On day 12, mice were euthanized, and tumors were collected, processed through a 100-μm strainer and stained for live/dead cell discrimination using the Zombie UV Fixable Viability Kit (BioLegend, 423108). Cells were blocked with FcR blocking reagent (Miltenyi Biotec, 130-092-575), and stained with fluorochrome-conjugated anti-mouse antibodies. Intracellular staining was performed after fixation and permeabilization (BD Biosciences, 554723), prior to the application of fluorochrome-conjugated anti-mouse antibodies. Data were analyzed on FlowJo. BMDM were incubated with medium alone, recombinant mouse IL-4 protein (Abcam, 9729) or LPS (Sigma, L2630-10MG), trypsinized with Trypsin-EDTA (0·05%) (Gibco, 25300120), washed, resuspended in FACS buffer (BD Pharmingen, 554657) and blocked with mouse FcR blocking reagent (Miltenyi Biotec, 130092575) on ice for 20 min. The cells were washed with FACs buffer, and stained for surface proteins 30 min at room temperature, fixed using True-Nuclear Fix Solution and their membranes were permeabilized using 1X True-nuclear Perm buffer (Biolegend, 424401). Cells were incubated with FcR blocking reagent again, followed by 30 min incubation time in room temperature, in the dark, for the antibody staining, and dead cells were excluded by adding Zombie UV fixable viability kit (Biolegend, 423107). Samples were analyzed with FlowJo software. For the compensation anti mouse compensation beads (Fisher Scientific, 501129040) were stained with each of the antibodies used, and calculated with the FACSDIVA software.

**Cell lines:** The mouse GBM CT2A cell line was provided by I. Verma (Salk Institute for Biological Studies, La Jolla, California, USA). TC1 cell line was previously generated and validated by Kanaya et al.(4) to represent NSCLC primary tumor and lung-to-leptomeningeal brain metastasis. UV2 cell line was provided by J. Sarkaria (Mayo Clinic, Rochester, Minnesota, USA). Cell lines were regularly tested for mycoplasma using a mycoplasma PCR kit (30-1012K, American Type Culture Collection [ATCC]). No additional cell line validation was performed by the authors.

**Antibodies and Nanobodies:** Catalog numbers for antibodies used for WB are presented in the relevant section. These antibodies were used based on manufacturers’ recommendations and relevant references. Nanobodies used in this study were generated and validated in house, with the process documented in this research paper. Antibodies used for flow cytometry are presented below along with their catalog numbers. Antibody dilutions recommended on the manufacturer’s website were further titrated by the authors and other lab members, to determine the appropriate working concentrations. Staining controls: unstained, live/dead staining alone and in combination with surface staining, extracellular staining without intracellular staining, and single-color control beads, which were used for compensation and gating strategies.

| Table: 1 T cell activation | | | | |  |
| --- | --- | --- | --- | --- | --- |
| Target | Manufacturer | | Catalog Number | |  |
| CD3 | BD Biosciences | | 563565 | |  |
| CD45 | Biolegend | | 103128 | |  |
| CD4 | BD Biosciences | | 563050 | |  |
| CD8 | Biolegend | | 100733 | |  |
| CD69 | Biolegend | | 104541 | |  |
| CD25 | BD Biosciences | | 552880 | |  |
| Tim3 | Biolegend | | 134019 | |  |
| LAG3 | Biolegend | | 125223 | |  |
| IFNg | Biolegend | | 507810 | |  |
| Table: 2 Macrophage polarization | | | | |  |
| Target | Manufacturer | | Catalog Number | |  |
| MHCII | Biolegend | | 107626 | |  |
| CD86 | Biolegend | | 159218 | |  |
| CD163 | Biolegend | | 155318 | |  |
| CSF1R | Biolegend | | 135529 | |  |
| PDL1 | Biolegend | | 155404 | |  |
| CD206 | Biolegend | | 141723 | |  |
| Table 3 csf1r functional assay | | | | | |
| Target | | Manufacturer | | Catalog Number | |
| CD3 | | BD Biosciences | | 563565 | |
| CD45 | | Biolegend | | 103128 | |
| F4/80 | | Biolegend | | 123147 | |
| CD11b | | Biolegend | | 101223 | |
| NOS2 | | eBioscience | | 12-5920-80 | |
| CD86 | | Biolegend | | 159218 | |
| CD206 | | Biolegend | | 141723 | |
| Table 4 Immune profiling | | | | | |
| Target | | Manufacturer | | Reference Number | |
| CD3 | | BD Biosciences | | 563565 | |
| CD45 | | Biolegend | | 103128 | |
| CD4 | | BD Biosciences | | 563050 | |
| CD8 | | Biolegend | | 100733 | |
| CD69 | | Biolegend | | 104541 | |
| LAG3 | | Biolegend | | 125223 | |
| TIM3 | | Biolegend | | 134019 | |
| F4/80 | | Biolegend | | 123147 | |
| CD11b | | BD Biosciences | | 561685 | |
| cd11c | | Biolegend | | 117347 | |
| xcr1 | | Biolegend | | 148225 | |
| cd86 | | Biolegend | | 159218 | |
| cd206 | | Biolegend | | 141723 | |
| mhcii | | Biolegend | | 107626 | |
| NOs2 | | eBioscience | | 12-5920-80 | |

**References**

1. Abramson J, Adler J, Dunger J, Evans R, Green T, Pritzel A, et al. Accurate structure prediction of biomolecular interactions with AlphaFold 3. Nature. 2024;630(8016):493-500.

2. Pettersen EF, Goddard TD, Huang CC, Meng EC, Couch GS, Croll TI, et al. UCSF ChimeraX: Structure visualization for researchers, educators, and developers. Protein Sci. 2021;30(1):70-82.

3. van Schaik TA, Moreno-Lama L, Aligholipour Farzani T, Wang M, Chen KS, Li W, et al. Engineered cell-based therapies in ex vivo ready-made CellDex capsules have therapeutic efficacy in solid tumors. Biomed Pharmacother. 2023;162:114665.

4. Kanaya N, Seddiq W, Chen KS, Kajiwara Y, Moreno Lama L, Borges P, et al. Engineered allogeneic stem cells orchestrate T lymphocyte-driven immunotherapy in immunosuppressive leptomeningeal brain metastasis. J Natl Cancer Inst. 2025;117(6):1151-65.
