# Supplementary material for: A nanobody-stem cell platform targeting innate and adaptive immune axis in the tumour microenvironment
Source: eBioMedicine. 2026 Jan 17;124:106122. doi: 10.1016/j.ebiom.2026.106122 (PMC12853783; doi:10.1016/j.ebiom.2026.106122)
Supplement: Supplementary Figures Caption [file mmc3.docx]

**Supplementary Figures**

**Supplementary Figure 1: Increased CSF-1R expression is related to worse overall survival and correlates with increased PD-1 expression levels.** A) Esophageal and Thymic Epithelial cancers overall survival (Mantel-Cox test). B, C) PD-1 expression in normal and melanoma and GBM, and correlation with CSF-1R expression, respectively (Student t-test). D) Comparison of levels of several key populations in low and high CSF-1R expression in GBM. *P < 0.05, **P < 0.01, ***P < 0.001, and ****P < 0.0001.

**Supplementary Figure 2: First selection steps from the pool of VHH clones against CSF1-R**. A) Periplasmic ELISA from the total number of clones. B) Receptor Occupancy from the most promising Nbs clones.

**Supplementary Figure 3: Nb-CSF1R70 and Nb-PD150 conjugation with AF647 and imaging of BMDM and T cells.** A, C) Showing the conjugation efficacy using a gel – Coomassie staining for the protein and AF647 for the conjugate. B, D) Staining on two different cells lines, using the Nbs conjugates. Scale bars apply to all the images in the same row, 50um.

**Supplementary Figure 4: Cell toxicity and cytotoxicity following incubation with PD1 Nbs**. A) Tumor viability post incubation with 100nM and 500nM BNbPD1 and AbPD1, n=4, significance using One-Way ANOVA where *P<0.05. B) T cell viability curves following 48hr incubation with varying Nbs concentrations (n=2).

**Supplementary Figure 5: Presence of exhaustion markers on T cell subsets following T cell activation and T cell viability in presence of Nbs against PD1.** A) Plots showing GFP expression of SC- BNb-PD1, SC-BNb-CSF1R. and SC-GFP. B) Dot blot analysis showing the presence of BNbPD1 and BNbCSF1R in the supernatant over 3 days. C) CD25+, LAG3+ and Tim3+ percentages on activated CD4+ and CD8+ T cells in presence of Nbs against PD1. For B and C significance was calculated with One-Way ANOVA with Tukey’s multiple comparisons test (*P < 0.05, **P < 0.01, ***P < 0.001, and ****P < 0.0001).

**Supplementary Figure 6: Prolonged secretion of biparatopic Nbs from SC.** A) Schematic description of the experimental plan and plots showing levels of commercial Abs targeting PD-1 and CSF-1R and BNbPD1 and BNbCSF1R in the tumor tissue, seven days post intravenous and intratumoral administration, respectively (n=3). B) H&E staining of 4 major organs in control (no SC) and experimental mice (with SC). Scale Bars apply to all the images in the same row, for brain, kidney and liver 1000um, for spleen 500um.

**Supplementary Figure 7: Raw uncropped WB.** A) uncropped WB showing the secretion of BNbCSF1R and BNbPD1 from engineered SCs. Red rectangles indicate the samples and bands shown in Figure 3B. B) Uncropped WB showing the secretion of BNbCSF1R and BNbPD1 from encapsulated engineered SCs over four days. Red rectangles indicate the samples and bands shown in Figure 6C. C) Uncropped Dotblot showing the sustained secretion of BNbCSF1R and BNbPD1 from engineered SCs over three days. Red rectangles indicate the samples and bands shown in Figure S3B.
