# Supplementary material for: A nanobody-stem cell platform targeting innate and adaptive immune axis in the tumour microenvironment
Source: eBioMedicine. 2026 Jan 17;124:106122. doi: 10.1016/j.ebiom.2026.106122 (PMC12853783; doi:10.1016/j.ebiom.2026.106122)

Uncropped Western Blots, Also presented in Supplementary Figure 7

A

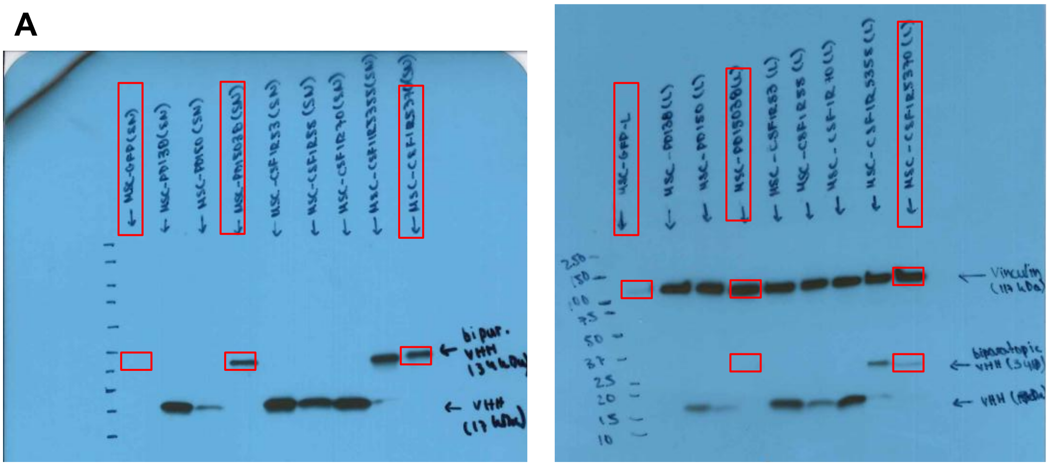

B

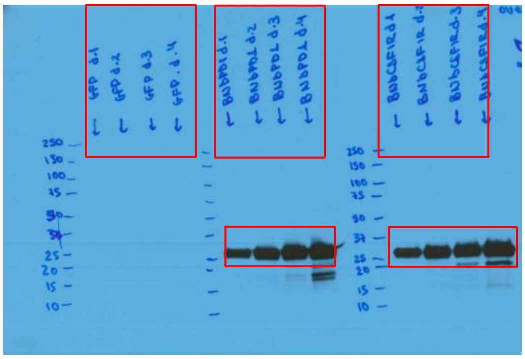

C

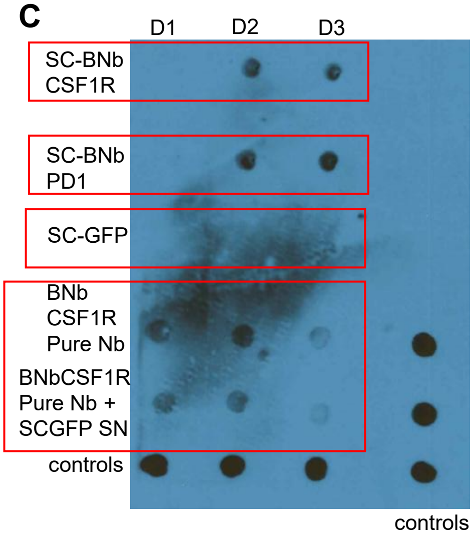

Supplement: Uncropped Western Blots [file mmc4.pdf]
